# Risk assessment for hospital admission in patients with COPD; a multi-centre UK prospective observational study

**DOI:** 10.1371/journal.pone.0228940

**Published:** 2020-02-10

**Authors:** Jilles M. Fermont, Charlotte E. Bolton, Marie Fisk, Divya Mohan, William Macnee, John R. Cockcroft, Carmel McEniery, Jonathan Fuld, Joseph Cheriyan, Ruth Tal-Singer, Ian B. Wilkinson, Angela M. Wood, Michael I. Polkey, Hana Müllerova

**Affiliations:** 1 Division of Experimental Medicine and Immunotherapeutics, Department of Medicine, University of Cambridge, Cambridge, England, United Kingdom; 2 British Heart Foundation Cardiovascular Epidemiology Unit, Department of Public Health and Primary Care, University of Cambridge, Cambridge, England, United Kingdom; 3 Division of Respiratory Medicine and NIHR Nottingham BRC Respiratory Theme, School of Medicine, University of Nottingham, Nottingham, England, United Kingdom; 4 Medical Innovation, Value Evidence and Outcomes GSK, Collegeville, PA, United States; 5 Centre for Inflammation Research, Queen’s Medical Research Institute, University of Edinburgh, Edinburgh, Scotland, United Kingdom; 6 Department of Cardiology, Columbia University Medical Centre, New York, New York, United States; 7 Department of Respiratory Medicine, Cambridge University Hospitals NHS Foundation Trust, Cambridge, England, United Kingdom; 8 Cambridge Clinical Trials Unit, Cambridge University Hospitals NHS Foundation Trust, Addenbrooke’s Hospital, Cambridge, England, United Kingdom; 9 British Heart Foundation Centre of Research Excellence, University of Cambridge, Cambridge, England, United Kingdom; 10 National Institute for Health Research Blood and Transplant Research Unit in Donor Health and Genomics, University of Cambridge, Cambridge, England, United Kingdom; 11 National Institute for Health Research Cambridge Biomedical Research Centre, University of Cambridge and Cambridge University Hospitals, Cambridge, England, United Kingdom; 12 Health Data Research UK Cambridge, Wellcome Genome Campus and University of Cambridge, Cambridge, England, United Kingdom; 13 Department of Respiratory Medicine, Royal Brompton Hospital, London, England, United Kingdom; 14 Epidemiology, Value Evidence and Outcomes GSK, Uxbridge, England, United Kingdom; National and Kapodistrian University of Athens, GREECE

## Abstract

In chronic obstructive pulmonary disease (COPD), acute exacerbation of COPD requiring hospital admission is associated with mortality and healthcare costs. The ERICA study assessed multiple clinical measures in people with COPD, including the short physical performance battery (SPPB), a simple test of physical function with 3 components (gait speed, balance and sit-to-stand). We tested the hypothesis that SPPB score would relate to risk of hospital admissions and length of hospital stay. Data were analysed from 714 of the total 729 participants (434 men and 280 women) with COPD. Data from this prospective observational longitudinal study were obtained from 4 secondary and 1 tertiary centres from England, Scotland, and Wales. The main outcome measures were to estimate the risk of hospitalisation with acute exacerbation of COPD (AECOPD and length of hospital stay derived from hospital episode statistics (HES). In total, 291 of 714 individuals experienced 762 hospitalised AECOPD during five-year follow up. Poorer performance of SPPB was associated with both higher rate (IRR 1.08 per 1 point decrease, 95% CI 1.01 to 1.14) and increased length of stay (IRR 1.18 per 1 point decrease, 95% CI 1.10 to 1.27) for hospitalised AECOPD. For the individual sit-to-stand component of the SPPB, the association was even stronger (IRR 1.14, 95% CI 1.02 to 1.26 for rate and IRR 1.32, 95% CI 1.16 to 1.49 for length of stay for hospitalised AECOPD). The SPPB, and in particular the sit-to-stand component can both evaluate the risk of H-AECOPD and length of hospital stay in COPD. The SPPB can aid in clinical decision making and when prioritising healthcare resources.

## Introduction

In the United Kingdom (UK), chronic obstructive pulmonary disease (COPD) related mortality and healthcare costs are projected to increase significantly, with annual direct healthcare costs in England alone expected to increase from £1.5 billion in 2011 to £2.32 billion by 2030.[1] Most of the cost of treating COPD arises from inpatient care,[2] specifically for hospitalised acute exacerbations (i.e. episodic worsening of symptoms) of COPD (H-AECOPD).

According to 2016–17 statistics of the National Health Services (NHS) Digital, more than 128,000 individuals with a specific code for COPD exacerbation in the United Kingdom were admitted to hospital, of which 97% were emergency admissions with a median hospital length of stay of three days.[3] Leaving aside cost, admission with AECOPD is a major event in the lifetime of a COPD patient. National UK audit data shows that, while outcomes have improved, in-patient mortality remains high (4.3%) with a further 2.8% of those discharged dying within 30 days. Importantly, longer length of stay was associated with increased mortality at both 30 and 90 days (9.9 and 22.6% respectively).[4]

The risk of having an acute exacerbation of COPD is determined by the patient’s contact with an infectious (i.e. bacterial or viral) or environmental (e.g. air pollution) triggers. However, the need for hospitalisation for AECOPD is also determined by the patient’s physical capacity. In a prior analysis of data from the COPD Biomarker Qualification Consortium,[5] we showed that an integrative test of physical capacity, the six minute walk test (6MWT) distance, was not at all predictive of the likelihood of acute exacerbation but was strongly predictive of one year mortality and to a lesser extent of hospitalisation.

The 6MWT is cumbersome in general practice and to some extent in secondary care; this is acknowledged in the recent update of the National Institute for Health and Care Excellence 2018 guidelines that recommended against the use of the BODE Index for prognostication since the components (which include 6MWT) are time-intensive and seldom feasible in primary care.[6] In particular, the 6MWT requires a minimum 30m flat, uninterrupted track in an unhurried setting, which is often unavailable, and when completed properly should be undertaken twice to minimise learning effect. Thus, a proper evaluation may take 30 minutes. In contrast, the short physical performance battery (SPPB) can be undertaken with no special facilities in a routine clinic and is used in clinical practice by geriatricians and those in pulmonary rehabilitation.[7]

We therefore hypothesised in this study that SPPB score could serve both to identify patients who are more likely to be hospitalised or, when admitted, to have a longer length of stay. Data from the UK multicentre Evaluating the Role of Inflammation in Chronic Airways disease (ERICA) cohort study, which has been described in more detail elsewhere[8,9] were used with the aim being to evaluate the relationship between functional measures, specifically SPPB and risk of H-AECOPD. Further, we aimed to determine a relationship with length of hospital stay for initial (i.e. first hospital admission after assessment) AECOPD. To address these questions, we combined routinely collected hospital electronic health record data with baseline ERICA data.

## Methods

### Study design and participants

Observational data is reported according to the STROBE statement.[10] Data were used from the ERICA cohort, a multi-centre observational, non-interventional, epidemiological study with a sample size of 729 individuals with stable COPD (Global Initiative for Chronic Obstructive Lung Disease grade II-IV). The study was originally designed and powered on the basis of a tertile analysis of variables pulse wave velocity and QMVC, based on an estimated sample size of 800 individuals with COPD. Full study design and participant details are available in the published ERICA cohort protocol.[8] The study is registered with the UK Clinical Research Network Study Portfolio (StudyID 11101). Baseline data were collected between December 2011 and January 2014. Patient level cohort data were linked to hospital admission data obtained from the NHS admitted patient care dataset, Hospital Episodes Statistics (HES) in England, Scotland and Wales, which captures all H-AECOPD, since cohort baseline visit until November 2017. Analyses were limited to a maximum five years of follow-up. See S1 Text for further details.

### Study outcomes

The primary outcome measure was H-AECOPD. These data were first cleaned for episode status and inpatient (i.e. hospitalised) AECOPD episodes were identified using validated criteria (S1 Table).[11] Acute exacerbations of COPD were extracted from both primary and secondary positions of international classification of diseases and related health problems 10^th^ revision coding. Only so-called definite and possible H-AECOPD were considered for this analysis. Only episodes during the study follow-up were evaluated. Admission and discharge dates were used to determine hospital length of stay (i.e. number of days) for initial H-AECOPD after ERICA assessment.

### Potential predictor variables

All significant variables reported by Hurst *et al*.[12] and functional measures assessed in the ERICA cohort were considered. A full list of predictor variables is shown in S2 Table including demographics, lung function measurements, blood markers, health-related quality of life and respiratory symptoms questionnaire data, and functional assessments. Measures of particular interest were those measuring physical capacity including SPPB and its components (i.e. 4-metre gait speed (4MGS), balance, and 5 repetition sit-to-stand), quadriceps maximum voluntary contraction (QMVC), and 6MWT. The SPPB is a battery of tests and used to evaluate the physical performance of the lower extremities. Its three components score 0–4. Total SPPB score is the sum of points of each component, with a maximum of twelve denoting no functional limitation. QMVC is a surrogate marker of functional activity (i.e. quadriceps muscle strength) where the best effort of six contractions was recorded, and the QMVC_%predicted_ was derived from the Seymour equation.[13] The 6MWT was used to assess exercise intolerance and to evaluate functional exercise capacity by recording the distance walked as quickly as possible for six minutes. The exacerbation history in the twelve months prior to study baseline was dichotomised (i.e. 0 vs. ≥ 1). Prior exacerbation history was self-reported and defined as requiring treatment with oral steroids or antibiotics.

### Statistical analysis

Missing values are described in S1 and S2 Figs, S2 Text; only complete cases were considered. Relationships between baseline variables were quantified using Spearman’s pair-wise correlations; values < 0.30 were considered weak, 0.30–0.50 as moderate, and > 0.50 as strong (S3 Fig).[14] Negative binomial regression was used to examine the association between functional measures and (i) the rate of H-AECOPD within the study period, and (ii) length of hospital stay (per day). Analyses were adjusted for exposure times (time between baseline visit date and earliest of death, or end of study period). Regression estimates are presented as incidence-rate ratios (IRR). Incidence risk ratios for log-transformed biomarkers represent a twofold increase in the biomarker.

All analyses were stratified by recruitment site and adjusted for age and sex. Further analyses were adjusted for body mass index, smoking status, and covariates found to be of significance in the main multivariate model by Hurst *et al*, namely exacerbation history (previous year), forced expiratory volume in one second (FEV_1_) measured in litres, and productive cough (defined using questionnaire data: “If you cough, do you produce phlegm (sputum)?”).[12] Covariates were tested for collinearity resulting in the omission of Medical Research Council dyspnoea score and white cell count. Predictors for the final analyses were derived sequentially, firstly estimating the association of each individual variable fully adjusted, following stepwise regression including the significant variables only, whilst considering collinearity and clinical utility.

In stepwise regression analysis, only predictors with a significance level above α 0.1 for backward selection and α 0.05 for forward stepwise selection were considered. For each stepwise regression, likelihood ratio tests were conducted to determine if independent variables should remain in the analysis or not, and the maximum number of variables considered in each regression analysis were based on the least number of events.[15] As sex and exacerbation history can act as effect modifiers, in sensitivity analyses, we explored analysis stratified by these factors and tested for interactions.

To evaluate the ability of SPPB, or its sit-to-stand component, to predict time to H-AECOPD, we used Cox-regression with significance assessed using log-rank test for trend. Estimates are displayed using Kaplan-Meier plots.

All tests were two-sided and of statistical significance level of p = 0.05. Our analyses were performed using Stata version 13 (College Station, Texas) and R (R Foundation).

## Results

### Descriptive statistics

In total, 714 individuals with stable COPD and complete data were included in the analysis. At baseline, the mean ± standard deviation age of the cohort was 67 ± 8 years with 61% of participants being male. A third of the cohort was overweight, another third obese; and a third were current smokers. Exacerbations during the year prior to baseline were self-reported by 67% individuals with a corresponding mean of 2 ± 2 events per person-year. Mean FEV_1_% predicted was 52 ± 16%. About half of the cohort (51%) experienced breathlessness that limited daily activities (Medical Research Council dyspnoea score ≥ 3) and 46% had productive cough on most mornings (Table 1 and S3 Table, S2 Text). Individuals with a history of AECOPD at baseline were more likely to be younger, female, have worse lung function, shorter walking distance, and lower SPPB scores.

**Table 1:** Baseline characteristics.

	Total	Without AECOPD history at baseline^a^	With AECOPD history at baseline ^a^	P value
Characteristic	**Mean ± SD or n (%)**			
**Description**				
Age (years)	67 ± 8	68 ± 8	67 ± 7	0.022
Sex, n (%)				< 0.001
Male	434 (61)	171 (72)	261 (55)	
Female	280 (39)	65 (28)	212 (45)	
Body mass index (kg/m^2^)	27 ± 6	27 ± 5	27 ± 6	0.389
**Musculoskeletal measures**				
6MWT distance (metre)	346 ± 130	384 ± 122	326 ± 130	< 0.001
SPPB (0–12)	10 ± 2	10 ± 2	9 ± 3	0.015
4MGS score (0–4)	4 ± 1	4 ± 1	3 ± 1	< 0.001
Balance points (0–4)	4 ± 1	4 ± 1	4 ± 1	0.719
Sit-to-stand score (0–4)	2 ± 1	3 ± 1	2 ± 1	0.004
QMVC peak (kg)	31 ± 11	29 ± 12	33 ± 11	< 0.001
QMVC % predicted	44 ± 8	46 ± 8	44 ± 8	0.005
**Lung function**				
FEV_1_% predicted	52 ± 16	57 ± 14	50 ± 16	< 0.001
Smoking status, n (%)				0.032
Current	218 (31)	85 (36)	133 (28)	
Former	492 (69)	151 (64)	340 (72)	
GOLD, n (%)				< 0.001
Grade II	406 (57)	166 (70)	237 (50)	
Grade III	240 (34)	56 (24)	183 (39)	
Grade IV	68 (10)	14 (6)	53 (11)	
Productive cough, n (%)				< 0.001
Never	46 (7)	32 (14)	14 (3)	
Other	662 (94)	200 (86)	459 (97)	
**Biochemical measures**				
log Glucose (mmol/L)	1.60 ± 0.15	1.61 ± 0.16	1.61 ± 0.15	0.945
log Fibrinogen (g/dL)	1.22 ± 0.23	1.18 ± 0.23	1.24 ± 0.23	0.002
log C-reactive protein (mg/L)	1.26 ± 1.08	1.11 ± 1.03	1.32 ± 1.09	0.016
GFR (mL/min/1.73 m^2^)	88 ± 18	87 ± 19	89 ± 18	0.443
Neutrophil count (mm^3^)	4.75 ± 1.70	4.57 ± 1.65	4.81 ± 1.67	0.015
**Cardiovascular status**				
Heart rate (bpm)	75 ± 13	74 ± 12	75 ± 12	0.546
**Questionnaires**				
SGRQ-C (0–100)	50 ± 21	40 ± 20	54 ± 20	< 0.001
CAT (0–40)	20 ± 8	17 ± 8	21 ± 8	< 0.001

Values are given as the mean and standard deviation, or No. of cases (%). Baseline data of study participants are included.

^a^Self-reported prior to study.

SD, standard deviation. 6MWT, six-minute walk test. SPPB, short physical performance battery. 4MGS, four-metre gait speed. QMVC, quadriceps maximum voluntary contraction. FEV_1_, forced expiratory volume in one second. GOLD, global initiative for obstructive lung disease. GFR, glomerular filtration rate. SGRQ-C, St George's respiratory questionnaire for COPD. CAT, COPD assessment test.

In total, 291 (41%) experienced at least one H-AECOPD during the study follow-up; 159 (22%) had multiple events (S4–S6 Figs). Overall, 127 (18%) individuals died and of these, the majority 103 (81%) died following discharge having been hospitalised for AECOPD during the study period. Median (interquartile range (IQR)) length of hospital stay for initial (i.e. first hospital admission after assessment) H-AECOPD was 3 (1–7) days. For the 159 readmitted, the median time to hospital readmission was 179 (54–421) days, of whom 65 individuals (41%) were readmitted to hospital within 90 days after initial admission and had a median length of stay of 3 (2–7) days.

### Factors associated with rate of H-AECOPD

**Fig 1:**
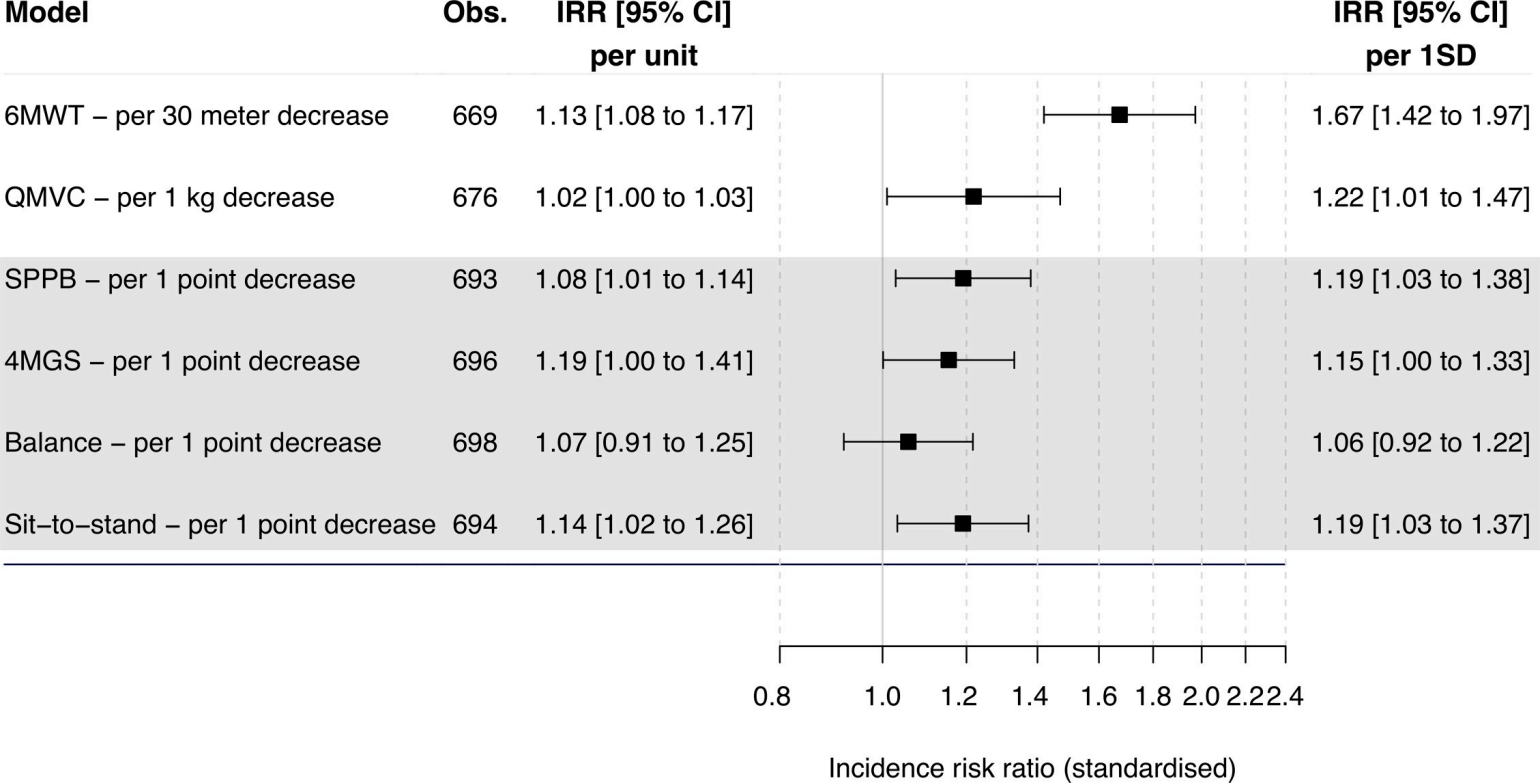
Associations of baseline musculoskeletal measures and rate of hospitalised acute exacerbation of chronic obstructive pulmonary disease in the ERICA cohort. Risk indicated as incidence risk ratios. Estimates derived using negative binomial regression. Analyses adjusted for recruitment site. Age, sex, body mass index, smoking status, forces expiratory volume in one second, phlegm, and exacerbation history were included as covariates. Figure displays standardised IRRs, allowing comparison of measurements on different scales. Obs, number of observations included in analysis. IRR, incidence risk ratios. CI, confidence intervals. SD, standard deviation. 6MWT, six-minute walk test. SPPB, short physical performance battery. 4MGS, four-metre gait speed. QMVC, quadriceps maximum voluntary contraction.

Adjusted analysis showed 6MWT, SPPB and its 4MGS, and sit-to-stand components, and QMVC were all associated with a higher risk of H-AECOPD (Fig 1 and S7 Fig, S4 Table). The 6MWT (IRR 1.13 per 30 metre decrease, 95% CI 1.08 to 1.17, p < 0.001), FEV_1_ (IRR 0.84 per 100 ml increase, 95% CI 0.81 to 0.86, p < 0.001) or disease severity measured by Global Initiative for Chronic Obstructive Lung Disease (IRR 2.51 per increase to next stage, 95% CI 2.04 to 3.10, p < 0.001), and males (IRR 2.41, 95% CI 1.77 to 3.29, p < 0.001) had the strongest associated IRRs.

Fully adjusted multivariable stepwise regression retained the following significant predictors: male gender (IRR 2.14, 95% CI 1.55 to 2.96, p < 0.001), FEV_1_, (IRR 0.88 per 100 ml increase, 95% CI 0.85 to 0.91, p < 0.001), exacerbation history ≥ 1 (IRR 1.96, 95% CI 1.39 to 2.76, p < 0.001), COPD assessment test score (IRR 1.03 per 1 point increase, 95% CI 1.01 to 1.05, p = 0.010), resting heart rate (IRR 1.01 per 1 bpm increase, 95% CI 1.00 to 1.03, p = 0.025), and 6MWT (IRR 1.08 per 30 metre decrease, 95% CI 1.04 to 1.12, p < 0.001; Table 2).

**Table 2:** Factors associated with rate of H-AECOPD in the stepwise multivariable model.

	Multivariable analysis^a^	Stepwise regression (n = 610)	
Factors	IRR (95% CI)	IRR (95% CI)	P value
Sex–male	2.41 (1.77 to 3.29)	2.14 (1.55 to 2.96)	< 0.001
6MWT distance–per 30 metre decrease	1.13 (1.08 to 1.17)	1.08 (1.04 to 1.12)	< 0.001
SPPB score–per 1 point decrease	1.08 (1.01 to 1.14)	Omitted	N/A
QMVC peak–per 1 kg decrease	1.02 (1.00 to 1.03)	Omitted	N/A
FEV_1_ –per 100 ml increase	0.84 (0.81 to 0.86)	0.88 (0.85 to 0.91)	< 0.001
Exacerbation history (1 year), ≥ 1^b^	1.94 (1.40 to 2.67)	1.96 (1.39 to 2.76)	< 0.001
Fibrinogen–per 1 log unit increase	1.95 (1.03 to 3.68)	Omitted	N/A
Neutrophils–per 1 unit increase	1.14 (1.05 to 1.24)	Omitted	N/A
Resting heart rate–per 1 bpm increase	1.02 (1.01 to 1.03)	1.01 (1.00 to 1.03)	0.025
SGRQ-C–per 4 point increase	1.07 (1.03 to 1.10)	Omitted	N/A
CAT–per 1 point increase	1.05 (1.03 to 1.07)	1.03 (1.01 to 1.05)	0.010

Factors significantly associated with rate of H-AECOPD were included in the stepwise regression. Analyses were adjusted for recruitment site.

^a^Adjusted for age, sex, body mass index, smoking status, forced expiratory volume in one second, phlegm, and exacerbation history.

^b^Self-reported prior to study.

IRR, incidence risk ratios. CI, confidence intervals. 6MWT, six-minute walk test. SPPB, short physical performance battery. QMVC, quadriceps maximum voluntary contraction. FEV_1_, forced expiratory volume in one second. SGRQ-C, St George's respiratory questionnaire for COPD. CAT, COPD assessment test.

### Factors associated with H-AECOPD length of stay

**Fig 2:**
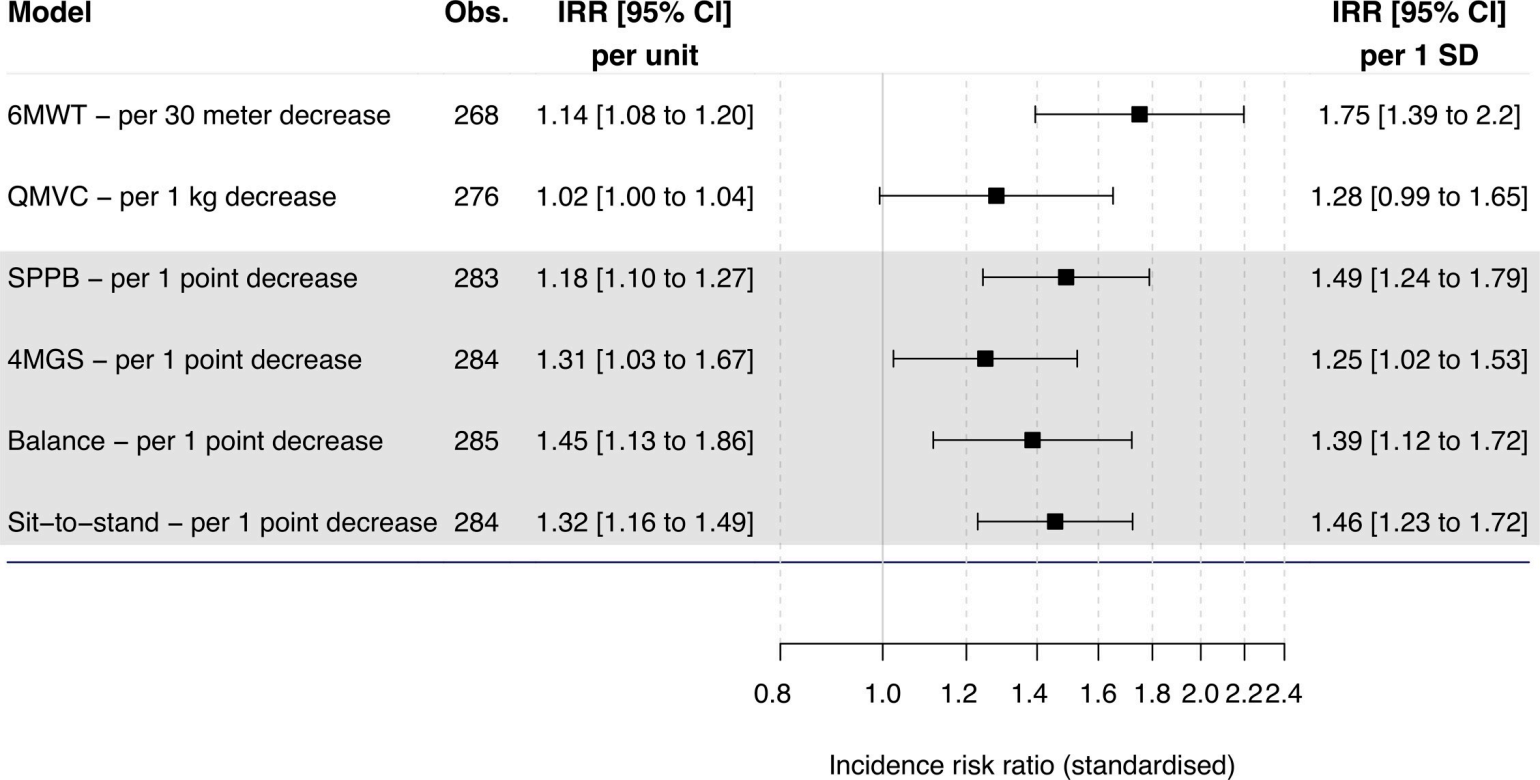
Associations of baseline musculoskeletal measures and hospital length of stay after admission for acute exacerbation of chronic obstructive pulmonary disease in the ERICA cohort. Risk indicated as incidence risk ratios. Estimates derived using negative binomial regression. Analyses adjusted for recruitment site. Age, sex, body mass index, smoking status, forces expiratory volume in one second, phlegm, and exacerbation history were included as covariates. Figure displays standardised IRRs, allowing comparison of measurements on different scales. Obs, number of observations included in analysis. IRR, incidence risk ratios. CI, confidence intervals. SD, standard deviation. 6MWT, six-minute walk test. SPPB, short physical performance battery. 4MGS, four-metre gait speed. QMVC, quadriceps maximum voluntary contraction.

Including data from individuals admitted to hospital only (n = 291), multivariable analysis identified multiple measures to be associated with H-AECOPD length of stay (Fig 2 and S8 Fig, S5 Table). All functional measures, except for QMVC were associated with a higher risk of H-AECOPD stay. Age (IRR 1.83 per 10-year increase, 95% CI 1.48 to 2.26, p < 0.001), 6MWT (IRR 1.14 per 30-metre decrease, 95% CI 1.08 to 1.20, p < 0.001), and SPPB (IRR 1.18 per 1 point decrease, 95% 1.10 to 1.27, p < 0.001) were the strongest associated variables.

Fully adjusted multivariable stepwise regression retained the following significant predictors: age (IRR 1.53 per 10-year increase, 95% CI 1.18 to 1.98, p = 0.001), body mass index (IRR 0.93 per 1 point increase, 95% CI 0.90 to 0.96, p < 0.001), glucose (IRR 2.89 per twofold increase, 95% CI 1.18 to 7.05, p = 0.020), and SPPB (IRR 1.19 per 1 point decrease, 95% CI 1.10 to 1.30, p < 0.001; Table 3).

**Table 3:** Factors associated with H-AECOPD length of stay in the stepwise multivariable model.

	Multivariable analysis^a^	Stepwise regression (n = 233)	
Factor	IRR (95% CI)	IRR (95% CI)	P value
Age–per 10 year increase	1.83 (1.48 to 2.26)	1.53 (1.18 to 1.98)	0.001
BMI–per 1 point increase	0.96 (0.93 to 0.99)	0.93 (0.90 to 0.96)	< 0.001
6MWT distance–per 30 metre decrease	1.14 (1.08 to 1.20)	Omitted	N/A
SPPB–per 1 point decrease^b^	1.18 (1.10 to 1.27)	1.19 (1.10 to 1.30)	< 0.001
Exacerbation history (1 year), ≥ 1^c^	0.62 (0.39 to 0.97)	Omitted	N/A
Glucose–per 1 log unit increase	8.78 (2.81 to 27.49)	2.89 (1.18 to 7.05)	0.020
Fibrinogen–per 1 log unit increase	3.14 (1.37 to 7.18)	Omitted	N/A
GFR–per 1 unit increase	0.98 (0.97 to 1.00)	Omitted	N/A

Factors significantly associated with H-AECOPD length of stay were included in the stepwise regression. Analyses were adjusted for recruitment site.

^a^Adjusted for age, sex, body mass index, smoking status, forced expiratory volume in one second, phlegm, and exacerbation history.

^b^When replacing SPPB with the sit-to-stand component both the sit-to-stand component and the 6MWT remain, but 6MWT is insignificant.

^c^Self-reported prior to study.

IRR, incidence risk ratios. CI, confidence intervals. BMI, body mass index. 6MWT, six-minute walk test. SPPB = short physical performance battery. GFR, glomerular filtration rate.

### Sensitivity analysis for rate of H-AECOPD

Overall, IRRs were higher for men and 6MWT for those with no exacerbation history (Table 2 and S6 Table). Incidence risk ratios of exacerbation history were higher for women when stratifying by gender (Table 2 and S7 Table). When testing for interactions, both prior exacerbation history and sex were significant.

**Fig 3:**
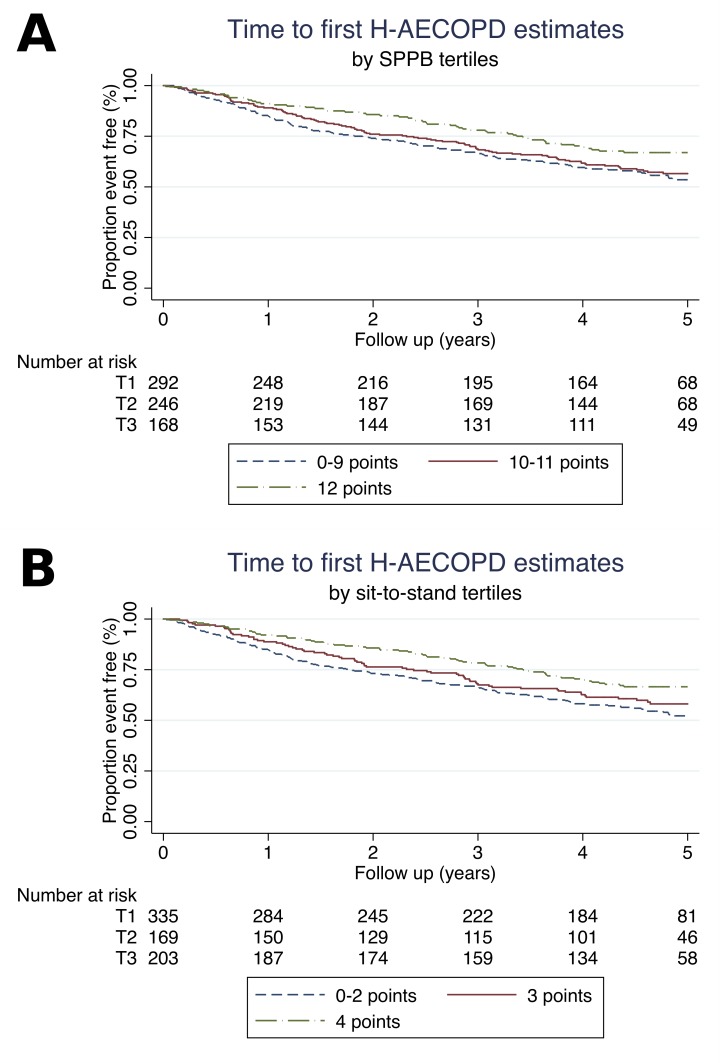
Time to first H-AECOPD estimates. (A) Estimates by SPPB tertiles. (B) Estimates by sit-to-stand tertiles.

Kaplan–Meier curves (Fig 3) according to SPPB tertiles demonstrated that reduced time to first H-AECOPD was associated with higher SPPB or sit-to-stand scores (log-rank test for trend: p = 0.032 and 0.008 respectively).

## Discussion

In patients with stable COPD, we show that assessments of physical function including the SPPB, and in particular the sit-to-stand component as a standalone, identify patients at risk of H-AECOPD. Furthermore, barring age, the sit-to-stand is the strongest associated measure that predicts length of stay for H-AECOPD. We additionally confirmed our prior findings, that 6MWT predicts H-AECOPD in individuals with COPD after adjusting for common and known predictive covariates. The only equipment required for the sit-to-stand test is a chair and a stopwatch (an integral function on most smartphones) it can be performed virtually anywhere, and the test can be performed in less than five minutes. Therefore we propose that it may be a useful tool in both primary and secondary care for identification of patients at risk of H-AECOPD as well as those that are likely to have a prolonged length of stay.

### Strengths of this study

This study has many strengths. Firstly, multiple functional measures were used simultaneously in a large cohort of individuals clinically stable at assessment. Thus, although the importance of physical performance has been previously reported for some of these assessments, this is the first study to compare several of these tests and also comprehensively control for all other clinical aspects of COPD. In addition, measurements were performed at five different sites across the UK.

Secondly, event rates were stable throughout the study period, which is not only encouraging but also rates were comparable to those in large cohort studies including ECLIPSE (Evaluation of COPD Longitudinally to Identify Predictive Surrogate End-points)[16] suggesting that the findings are generalisable outside the UK. Hospital admissions were identified using validated criteria, and only definite and possible episodes were included in the analysis. In contrast to self-reported hospital admission, which may suffer from under-reporting,[17,18] H-AECOPD episodes were captured objectively using electronic health record data. Individuals had different observation periods as a result of different entry times into the study. The use of study inclusion and admission (i.e. event) dates allowed to adjust for exposure time and therefore used the correct probability distributions.

Third, while previous exacerbation is an established predictor of exacerbation that can be easily assessed, we also investigated factors associated with hospitalisation in patients without exacerbation history. This is of interest since in patients with no previous exacerbation, the predictors of an exacerbation are less well established. Lastly, collection of a comprehensive data set permitted better adjustment for covariates than prior single centre studies.

### Limitations of this study

Naturally this study has several limitations. Hospital Episode Statistics were obtained from the NHS Digital (England), NHS Scotland and NHS Wales. Apart from admission and discharge dates, we did not have spell data (i.e. total continuous stay and use of a hospital bed) available for individuals registered with the NHS Scotland and NHS Wales. The study period covered the time from study enrolment until the end of study, or death, and therefore the pre-enrolment hospital use history could not be obtained from HES. The final number of individuals included in the analysis were limited to those followed up by the NHS, slightly reducing the statistical power.

The study size precluded stratification by gender to assess the association between baseline measures and H-AECOPD stay (i.e. duration). Also, we explored for non-linearity of variables considered but lacked statistical power to identify any difference. There were differences in study populations between recruitment sites. For example, individuals from Cardiff were more symptomatic (based on COPD impact scores); however we caution that the departments at each of the five participating hospitals had variations in practice making analysis of difference in prognosis between sites of doubtful value. Nevertheless, we addressed this by adjusting for recruitment site in our analyses. There were missing data; in order to optimise the analysis, we included as many observations as possible and reported the number of observations included in each analysis.

Analyses were also adjusted for productive cough, believed to be an indicator of inflammation. A large proportion had productive cough on most mornings but there was no significant association with the outcomes in our cohort (see supplementary tables).

Quadriceps maximum voluntary contraction was included in the study both because we have previously found it to predict survival in COPD[19] and also because mechanistically it could explain SPPB score.[20] The study did not confirm QMVC to be a measure that could have widespread uses despite being associated with a higher risk of H-AECOPD, since it was not related to length of hospital stay. In addition, the test requires specialist equipment that is bulky and not currently commercially available. However, the effect of the SPPB, and therefore also QMVC, is potentially underestimated due to the relatively high SPPB mean score (10 points), with scores above ten indicating no functional limitation.

### Meaning of this study

Our data suggest that the SPPB performed almost as well as the 6MWT for risk of H-AECOPD while being more practical; moreover the sit-to-stand component in particular could be useful as a standalone test in time-limited settings such as primary care with confidence since it was not inferior to the SPPB. The good performance of SPPB was not entirely unexpected. In the geriatrics literature, the SPPB is a well-established tool for predicting risk of admission to nursing home facilities. In a prior study, Kon *et al*. showed that one component of SPPB, the 4MGS, when measured at point of discharge for H-AECOPD, had a strong predictive value for 90-day readmission however that population has a strong pre-test probability of admission.[21] In a similar population, Barker *et al*. reported that overall SPPB measured at point of discharge following AECOPD was predictive of mortality risk.[22] In a study of 50 COPD patients the sit-to-stand performance related to other prognostic scoring systems in COPD, including the BODE score.[23]. In addition, the sit-to-stand component of the SPPB battery has some similarities with the one minute sit-to-stand test which is predictive of mortality in COPD.[24,25] However, the present study extends knowledge by showing that that the sit-to-stand and the SPPB are associated both with admission risk and length of stay in stable COPD outpatients, which is important for primary and secondary care. Importantly, no other study provides data suggesting that in patients with stable COPD, SPPB or the sit-to-stand are associated with H-AECOPD incidence as well as related length of stay, and this information further adds support for SPPB being used as a drug development tool and endpoint for clinical trials addressing AECOPD, especially since the European Medicines Agency favours the SPPB as the measure of choice in the assessment of frailty.[26] More recently, Hopkinson *et al*. emphasised the importance of considering individual risk factors such as exercise capacity, which influences long term prognosis including hospital admission.[27]

Some treatments are presently available that can reduce the risk of exacerbation including vaccination and optimisation of inhaled therapies, which it is increasingly recognised, should be tailored to the individuals eosinophil status.[28] In addition, exercise-based treatments, most notably pulmonary rehabilitation, can increase physical capacity.[29,30] Lastly although the available data are mixed, some reports suggest that, at least in subsets of COPD patients, novel strategies to deliver pulmonary rehabilitation can reduce re-admission rates and length of stay by early application of telemedicine techniques.[31,32] All of these interventions have costs and thus in terms of prioritising patients who will derive most benefit there is a need for a stratification tool. Based on our findings we propose that the sit-to-stand component be adopted as a routine measure in the care pathway for COPD patients, as its use can aid in identification of at-risk patients and thus aid resource planning.

### Future studies

Future studies, using larger cohorts and/or different geographical populations, should replicate our findings. In particular, when developing or evaluating a multivariable prognostic model, the sample size should be estimated based on the *D* or *C-*statistic in order to sufficiently capture the significance of prognostic of specific biomarkers and produce robust estimates.[33] In addition, evaluating these measures longitudinally would allow better estimation of the association between H-AECOPD rate, duration, and readmission at different time points, as well as estimation of a minimally important difference in COPD, the latter of which is currently lacking in literature.

## Conclusions

Findings indicate that physical function including the SPPB, and in particular the sit-to-stand component as a standalone test can both evaluate the risk of H-AECOPD and length of hospital stay in individuals with COPD. Moreover since sit-to-stand and SPPB can be performed almost anywhere and without special equipment. Our data support the use of sit-to-stand or SPPB to aid in clinical decision making at an individual level and when prioritising healthcare resources, and support the incorporation of this tool into the annual COPD patient clinical review.

## Supporting information

S1 Text Study details. (DOCX)

S2 Text Baseline univariate analysis results. (DOCX)

S1 Table ICD-10 codes to ascertain acute exacerbation in COPD in the hospital episode statistics. (DOCX)

S2 Table List of covariates considered. (DOCX)

S3 Table Baseline characteristics of the ERICA cohort, by recruitment centre. (DOCX)

S4 Table Adjusted multivariable associations with rate of H-AECOPD. (DOCX)

S5 Table Adjusted multivariable associations with H-AECOPD length of stay. (DOCX)

S6 Table Adjusted multivariate associations with H-AECOPD rate, by exacerbation history. (DOCX)

S7 Table Adjusted multivariate associations with H-AECOPD rate, by sex. (DOCX)

S1 Fig Percentage of missing values. GFR = glomerular filtration rate. 6MW = six-minute walk. QMVC = quadriceps maximum voluntary contraction. CRP = C-reactive protein. HR = heart rate. CAT = COPD assessment test. SPPB = short physical performance battery. BMI = body mass index. FEV1 = forced expiratory volume in one second. GOLD = global initiative for obstructive lung disease.
(PDF)

S2 Fig Missing data patterns. SGRQ = St. George respiratory questionnaire for COPD. QMVC = quadriceps maximum voluntary contraction. 6MW = six-minute walk. GFR = glomerular filtration rate.
(PDF)

S3 Fig Spearman’s pair-wise correlations of baseline variables. EXAC = exacerbation history. MRC = Medical Research Council dyspnoea score. SGRQ = St. George respiratory questionnaire for COPD. CAT = COPD assessment test. HR = heart rate. WBC = white cell count. NEUT = neutrophils. FIB = fibrinogen. CRP = C-reactive protein. CHOL = total cholesterol. SMOKE = smoking status. PHL = phlegm. GRF = glomerular filtration rate. HB = haemoglobin. WD = six-minute walk. SPPB = short physical performance battery. FEV = forced expiratory volume in one second. QMVC = quadriceps maximum voluntary contraction. BMI = body mass index. BG = glucose. Correlation coefficients with a values <0.30 were considered weak, 0.30–0.50 as moderate, and >0.50 as strong.
(PDF)

S4 Fig Participant enrolment flow diagram for up to five years follow up of H-AECOPD. Total number of H-AECOPD (n = 291). FEV1 = forced expiratory volume in one second. FVC = forced vital capacity. NHS = National Health Services. H-AECOPD = hospitalised acute exacerbation of COPD. FUP = follow-up period.
(DOCX)

S5 Fig Type of H-AECOPD, by recruitment centre. Hospital admission data obtained from the National Health Service (NHS) Digital, NHS Wales, and NHS Scotland.
(PDF)

S6 Fig Yearly event rates, frequency and duration of H-AECOPD. (A) Mean event rates with 95% confidence intervals per 100 per-years during study period, (B) H-AECOPD frequency, and (C) H-AECOPD duration. Depth of blue indicates the cumulative number of individuals with first H-AECOPD during the study period. Red dashed line indicates the median number of hospital admissions for H-AECOPD amongst those experienced an H-AECOPD.
(PDF)

S7 Fig Associations of baseline measures and rate of H-AECOPD in the ERICA cohort. (PDF)

S8 Fig Associations of baseline measures and H-AECOPD length of stay in the ERICA cohort. (PDF)
