# Supplementary material for: Risk assessment for hospital admission in patients with COPD; a multi-centre UK prospective observational study
Source: PLoS One. 2020 Feb 10;15(2):e0228940. doi: 10.1371/journal.pone.0228940 (PMC7010290; doi:10.1371/journal.pone.0228940)
Supplement: S1 Text — (DOCX) [file pone.0228940.s001.docx]

**S1 Text. Study details.**

Baseline data were collected between December 2011 and January 2014. Demographic, clinical and patient reported measures and biomarkers including musculoskeletal measures were collected at baseline. Prior exacerbation history was defined as self-reported antibiotics and/or steroids use in the previous year (twelve months before baseline). Body mass index was categorised according to the World Health Organization.^[[1]](#footnote-1)^ Disease severity was defined by global initiative for obstructive lung disease (GOLD) stage and estimated as described by the GOLD.^[[2]](#footnote-2)^ Productive cough (i.e. phlegm) was defined using questionnaire data and considered a surrogate marker of inflammation. Where there was missing data for phlegm, data from the phlegm question of the St. George Respiratory Questionnaire for COPD (SGRQ-C), and the COPD Assessment Test (CAT) were used. Phlegm was dichotomised (never vs. other). Analyses were stratified by recruitment site, and adjusted for age, sex. Further analyses were adjusted for body mass index, smoking status, and covariates found to be of significance in the main multivariate model by Hurst *et al.*,^[[3]](#footnote-3)^ namely exacerbation history (previous year), forced expiratory volume in one second (FEV_1_) measured in litres, and phlegm. Covariates were tested for collinearity resulting in the omission of Medical Research Council (MRC) dyspnoea score and white cell count.

The mean number of H-AECOPD were 1.3 vs. 0.6 with a variance of 6.2 vs. 2.6 for those with an exacerbation history compared to those without, indicating over-dispersed count data.

1. World Health Organisation. The International Classification of adult underweight, overweight and obesity according to BMI. WHO; 2016. [↑](#footnote-ref-1)
2. Global Initiative for Chronic Obstructive Lung Disease (GOLD). Global Strategy for the Diagnosis, Management and Prevention of COPD, 2016. [↑](#footnote-ref-2)
3. Hurst JR *et al.* Susceptibility to exacerbation in chronic obstructive pulmonary disease. The New England journal of medicine 2010; 363(12): 1128-38. [↑](#footnote-ref-3)
