# Supplementary material for: Risk assessment for hospital admission in patients with COPD; a multi-centre UK prospective observational study
Source: PLoS One. 2020 Feb 10;15(2):e0228940. doi: 10.1371/journal.pone.0228940 (PMC7010290; doi:10.1371/journal.pone.0228940)
Supplement: S2 Text — (DOCX) [file pone.0228940.s002.docx]

**S2 Text. Baseline univariate analysis results.**

Those with missing values for 6MWT distance (n = 31) had a higher rate of H-AECOPD (p = 0.047). Those with an exacerbation history at baseline were younger (p = 0.027), male (p < 0.001), had lower forced expiratory volume in one second (p < 0.001), worse dyspnoea scores (p = 0.002), and higher inflammatory levels of fibrinogen (p = 0.001) and C-reactive protein (p = 0.019) compared to those without. Shorter walking distance (p < 0.001), lower short physical performance battery scores (p = 0.003), or the components four-metre gait speed (p <0.001) and chair stand (p = 0.003) but not balance (p = 0.630), and quadriceps maximum voluntary contraction (p < 0.001) were also reported for those with an exacerbation history at baseline.
