# Supplementary material for: Risk assessment for hospital admission in patients with COPD; a multi-centre UK prospective observational study
Source: PLoS One. 2020 Feb 10;15(2):e0228940. doi: 10.1371/journal.pone.0228940 (PMC7010290; doi:10.1371/journal.pone.0228940)
Supplement: S1 Table — (DOCX) [file pone.0228940.s003.docx]

**S1 Table. ICD-10 codes to ascertain acute exacerbation in COPD in the hospital episode statistics.**

| End point | ICD-10 codes | Disease/  Category | Use to ascertain AECOPD usage |
| --- | --- | --- | --- |
| J22 | Lower respiratory tract infection | Possible | Use if COPD diagnosed in primary care data in First position of any finished consultant episode |
| J41 | Simple and mucopurulent chronic bronchitis | Possible | Use if COPD diagnosed in primary care data in First position of any finished consultant episode |
| J41.0 | Simple chronic bronchitis | Possible | Use if COPD diagnosed in primary care data in First position of any finished consultant episode |
| J41.1 | Mucopurulent chronic bronchitis | Possible | Use if COPD diagnosed in primary care data in First position of any finished consultant episode |
| J41.8 | Mixed simple and mucopurulent chronic bronchitis | Possible | Use if COPD diagnosed in primary care data in First position of any finished consultant episode |
| J42 | Unspecified chronic bronchitis | Possible | Use if COPD diagnosed in primary care data in First position of any finished consultant episode |
| J43 | Emphysema | Possible | Use if COPD diagnosed in primary care data in First position of any finished consultant episode |
| J43.0 | MacLeod's syndrome | Possible | Use if COPD diagnosed in primary care data in First position of any finished consultant episode |
| J43.1 | Panlobular emphysema | Possible | Use if COPD diagnosed in primary care data in First position of any finished consultant episode |
| J43.2 | Centrilobular emphysema | Possible | Use if COPD diagnosed in primary care data in First position of any finished consultant episode |
| J43.8 | Other emphysema | Possible | Use if COPD diagnosed in primary care data in First position of any finished consultant episode |
| J43.9 | Emphysema, unspecified | Possible | Use if COPD diagnosed in primary care data in First position of any finished consultant episode |
| J44 | Other chronic obstructive pulmonary disease | Possible | Use if COPD diagnosed in primary care data in First position of any finished consultant episode |
| J44.0 | Chronic obstructive pulmonary disease with acute lower respiratory infection | Definite | Any position of any finished consultant episode as per validation study |
| J44.1 | Chronic obstructive pulmonary disease with acute exacerbation, unspecified | Definite | Any position of any finished consultant episode as per validation study |
| J44.8 | Other specified chronic obstructive pulmonary disease | Possible | Ditto |
| J44.9 | Chronic obstructive pulmonary disease, unspecified | Possible | First position of any finished consultant episode as per validation study |
| J45 | Asthma | Potential | If increased sensitivity required, use if COPD diagnosed in primary care data in First position of any finished consultant episode |
| J45.0 | Predominantly allergic asthma | Potential | If increased sensitivity required, use if COPD diagnosed in primary care data in First position of any finished consultant episode |
| J45.1 | Nonallergic asthma | Potential | If increased sensitivity required, use if COPD diagnosed in primary care data in First position of any finished consultant episode |
| J45.8 | Mixed asthma | Potential | If increased sensitivity required, use if COPD diagnosed in primary care data in First position of any finished consultant episode |
| J45.9 | Asthma, unspecified | Potential | If increased sensitivity required, use if COPD diagnosed in primary care data in First position of any finished consultant episode |
| J45 | Asthma | Potential | If increased sensitivity required, use if COPD diagnosed in primary care data in First position of any finished consultant episode |
| J46 | Status asthmaticus | Potential | If increased sensitivity required, use if COPD diagnosed in primary care data in First position of any finished consultant episode |
| J47.0 | Bronchiectasis with acute lower respiratory infection | Potential | If increased sensitivity required, use if COPD diagnosed in primary care data in First position of any finished consultant episode |
| J47.1 | Bronchiectasis with (acute) exacerbation | Potential | If increased sensitivity required, use if COPD diagnosed in primary care data in First position of any finished consultant episode |
| J47.9 | Bronchiectasis, uncomplicated | Potential | If increased sensitivity required, use if COPD diagnosed in primary care data in First position of any finished consultant episode |
| J96.0 | Acute respiratory failure | Potential | If increased sensitivity required, use if COPD diagnosed in primary care data in First position of any finished consultant episode |
| J96.2 | Acute and chronic respiratory failure | Potential | Use to ascertain AECOPD usage |

ICD-10 = international disease classification tenth edition. AECOPD = acute exacerbation of chronic obstructive pulmonary disease.
