# Supplementary material for: Risk assessment for hospital admission in patients with COPD; a multi-centre UK prospective observational study
Source: PLoS One. 2020 Feb 10;15(2):e0228940. doi: 10.1371/journal.pone.0228940 (PMC7010290; doi:10.1371/journal.pone.0228940)
Supplement: S2 Table — (DOCX) [file pone.0228940.s004.docx]

**S2 Table. List of covariates considered.**

| **Demographics** | **Lung function** | **Biochemical measures** | **Cardiovascular status** | **Questionnaires** | **Musculoskeletal measures** |
| --- | --- | --- | --- | --- | --- |
| Age | FEV_1_ | Glucose | Resting heart rate | SGRQ-C | 6MWT distance |
| Sex | Smoking status | Fibrinogen |  | CAT | SPPB  - 4MGS  - Balance  - Chair stand |
| BMI | Exacerbation history | CRP |  |  | QMVC |
|  | Phlegm | GFR |  |  |  |
|  |  | Neutrophils |  |  |  |
|  |  | Haemoglobin |  |  |  |
|  |  | Total cholesterol |  |  |  |

White cell count and Medical Research Council (MRC) dyspnoea score were omitted due to collinearity with musculoskeletal measures. BMI = body mass index. FEV_1_ = forced expiratory volume in one second. CRP = C-reactive protein. GFR = glomerular filtration rate. SGRQ-C = St. George respiratory questionnaire for COPD. CAT = COPD assessment test. 6MWT = six-minute walk test. SPPB = short physical performance battery. 4MGS = four-metre gait speed. QMVC = quadriceps maximum voluntary contraction.
