# Supplementary material for: Risk assessment for hospital admission in patients with COPD; a multi-centre UK prospective observational study
Source: PLoS One. 2020 Feb 10;15(2):e0228940. doi: 10.1371/journal.pone.0228940 (PMC7010290; doi:10.1371/journal.pone.0228940)
Supplement: S3 Table — (DOCX) [file pone.0228940.s005.docx]

**S3 Table. Baseline characteristics of the ERICA cohort, by recruitment centre.**

| Characteristic | Total (n = 714) | Site 1 (n = 86) | Site 2 (n = 97) | Site 3 (n = 370 | Site 4 (n = 102) | Site 5 (n = 59) | *P* value ^b^ |
| --- | --- | --- | --- | --- | --- | --- | --- |
| **Description** |  |  |  |  |  |  |  |
| Age (years) | 67 (62-73) | 69 (65-74) | 69 (64-75) | 67 (62-73) | 68 (62-72) | 65 (60-72) | 0.027 |
| Sex - male, n (%) | 434 (61) | 67 (78) | 57 (59) | 197 (53) | 73 (72) | 40 (68) | < 0.001 |
| Body mass index (kg/m^2^) | 27 (23-31) | 26 (23-30) | 26 (23-29) | 28 (24-32) | 27 (22-31) | 23 (20-27) | < 0.001 |
| **Lung function** |  |  |  |  |  |  |  |
| FEV_1_ (litre) | 53 (40-65) | 53 (35-61) | 53 (41-66) | 54 (42-66) | 54 (44-63) | 40 (27-60) | < 0.001 |
| Smoking status, n (%)  Current | 218 (31) | 14 (16) | 31 (32) | 137 (37) | 29 (29) | 7 (12) | < 0.001 |
| MRC dyspnoea score, ≥ 2, n (%) | 646 (91) | 75 (88) | 92 (95) | 345 (94) | 81 (80) | 53 (93) | < 0.001 |
| GOLD, n (%)  Stage IV | 68 (10) | 13 (15) | 3 (3) | 28 (8) | 5 (5) | 19 (32) | < 0.001 |
| Exacerbation history (1 year), ≥ 1 | 473 (67) | 60 (71) | 64 (66) | 242 (65) | 68 (67) | 39 (68) | 0.871 |
| Productive cough – yes | 327 (46) | 30 (35) | 37 (38) | 177 (48) | 56 (55) | 27 (47) | 0.034 |
| **Biochemical measures** |  |  |  |  |  |  |  |
| log Glucose (mmol/L) | 1.59 (1.50-1.69) | 1.55 (1.48-1.65) | 1.59 (1.53-1.67) | 1.59 (1.53-1.69) | 1.63 (1.55-1.70) | 1.55 (1.46-1.63) | < 0.001 |
| log Fibrinogen (g/dL) | 1.22 (1.06- 1.36) | 1.13 (1.03-1.22) | 1.25 (1.10-1.34) | 1.22 (1.06-1.39) | 1.25 (1.13-1.44) | 1.24 (1.10-1.36) | 0.001 |
| log C-reactive protein (mg/L) | 1.21 (0.47-2.01) | 1.10 (0.40-1.83) | 1.41 (0.69-2.21) | 1.18 (0.48-2.04) | 1.20 (0.36-2.06) | 1.39 (0.00-1.95) | 0.470 |
| log WCC (mcL) | 1.96 (1.79-2.15) | 1.95 (1.79-2.10) | 2.00 (1.82-2.19) | 1.96 (1.79-2.14) | 2.01 (1.81-2.23) | 1.96 (1.84-2.20) | 0.305 |
| GFR (mL/min/1.73 m^2^) | 87.2 (76.5-101.0) | 77.6 (63.6-90.2) | 90.2 (77.8-100.3) | 91.7 (80.7-103.7) | 78.6 (70.1-88.2) | 98.6 (81.4-113.5) | < 0.001 |
| Neutrophil count (mm^3^) | 4.5 (3.6-5.6) | 4.5 (3.8-5.5) | 4.7 (3.8-5.8) | 4.3 (3.5-5.4) | 4.8 (3.6-5.8) | 4.4 (3.4-5.9) | 0.136 |
| Haemoglobin (g/dL) | 14.3 (13.4 -15.3) | 14.4 (13.5-15.0) | 14.7 (13.8-15.5) | 14.1 (13.05-15.2) | 14.8 (13.8-15.9) | 14.3 (13.5-15.0) | < 0.001 |
| Total cholesterol (mmol/L) | 5.0 (4.3-5.8) | 5.1 (4.2-5.9) | 5.0 (4.4-5.7) | 5.0 (4.3-5.9) | 5.05 (4.4-5.9) | 4.9 (4.3-5.6) | 0.9385 |
| **Cardiovascular status** |  |  |  |  |  |  |  |
| Heart rate (bpm) | 74 (66-82) | 74 (67-83) | 72 (64-79) | 73 (66-81) | 78 (67-86) | 79 (68-87) | 0.015 |
| **Questionnaires** |  |  |  |  |  |  |  |
| SGRQ-C (0-100) | 51 ((34-66) | 42 (29-58) | 48 (32-60) | 55 (37-71) | 50 (32-63) | 50 (36-63) | < 0.001 |
| CAT (0-40) | 20 (13-26) | 16 (12-22) | 19 (13-25) | 21 (15-27) | 20 (14-25) | 18 (12-24) | < 0.001 |
| **Musculoskeletal measures** |  |  |  |  |  |  |  |
| 6MW distance (metre) | 366 (255-440) | 420 (300-498) | 399 (298-480) | 346 (220-420) | 363 (255-436) | 423 (302-480) | < 0.001 |
| SPPB score (0-12) | 10 (8-11) | 11 (10-12) | 11 (9-11) | 9 (7-11) | 11 (9-12) | 11 (10-12) | < 0.001 |
| QMVC peak (kg) | 30 (22-39) | 31 (26-40) | 30 (24-38) | 28 (20-37) | 33 (25-42) | 31 (23-36) | < 0.001 |
| Event rate, per 100 person-years (95% CI) | 11 (10-13) | 10 (7-15) | 16 (12-21) | 10 (8-12) | 9 (7-13) | 20 (14-27) | ~ |

Values are given as the median and interquartile range (IQR), or No. of cases (%). Baseline data of 714 patients are included.

FEV_1_ = forced expiratory volume in one second. MRC = Medical Research Council. GOLD = global initiative for obstructive lung disease. WCC = white cell count. GFR = glomerular filtration rate. SGRQ-C = St. George respiratory questionnaire for COPD. CAT = COPD assessment test. 6MWT = six-minute walk test. SPPB = short physical performance battery. QMVC = quadriceps maximum voluntary contraction.
