# Supplementary material for: Risk assessment for hospital admission in patients with COPD; a multi-centre UK prospective observational study
Source: PLoS One. 2020 Feb 10;15(2):e0228940. doi: 10.1371/journal.pone.0228940 (PMC7010290; doi:10.1371/journal.pone.0228940)
Supplement: S4 Table — (DOCX) [file pone.0228940.s006.docx]

**S4 Table. Adjusted multivariable associations with rate of H-AECOPD.**

|  | 5 year (n = 291 individuals with H-AECOPD) | | | |
| --- | --- | --- | --- | --- |
| **Baseline Characteristics** | **Incidence risk ratio (95% CI) ^a^** | ***P* value ^c^** | **Incidence risk ratio (95% CI) ^b^** | ***P* value ^c^** |
| **Description** |  |  |  |  |
| Age – per 10 year increase | 0.97 (0.81 to 1.17) | 0.773 | 0.88 (0.74 to 1.04) | 0.126 |
| Sex – male | 1.01 (0.74 to 1.39) | 0.932 | 2.41 (1.77 to 3.29) | < 0.001 |
| Body mass index – per 1 point increase | 0.96 (0.94 to 0.98) | 0.002 | 1.00 (0.98 to 1.02) | 0.947 |
| **Lung function** |  |  |  |  |
| FEV_1_ – per 100 ml increase | 0.83 (0.80 to 0.85) | < 0.001 | 0.84 (0.81 to 0.86) | < 0.001 |
| Smoking status – current | 1.25 (0.89 to 1.76) | 0.192 | 1.15 (0.84 to 1.57) | 0.382 |
| GOLD stage – per increase to next stage | 2.71 (2.21 to 3.33) | < 0.001 | 2.51 (2.04 to 3.10) | < 0.001 |
| Exacerbation history (1 year), ≥ 1 | 2.52 (1.79 to 3.53) | < 0.001 | 1.94 (1.40 to 2.67) | < 0.001 |
| Productive cough – yes | 1.90 (1.00 to 3.61) | 0.049 | 1.04 (0.79 to 1.38) | 0.768 |
| **Biochemical measures** |  |  |  |  |
| Glucose – per 1 log unit increase | 1.43 (0.53 to 3.87) | 0.477 | 1.77 (0.69 to 4.53) | 0.231 |
| Fibrinogen – per 1 log unit increase | 3.43 (1.71 to 6.88) | 0.001 | 1.95 (1.03 to 3.68) | 0.040 |
| CRP – per 1 log unit increase | 1.18 (1.03 to 1.35) | 0.018 | 1.10 (0.98 to 1.25) | 0.116 |
| GFR – per 1 unit increase | 1.01 (1.00 to 1.02) | 0.093 | 1.00 (0.99 to 1.01) | 0.621 |
| Neutrophils – per 1 unit increase | 1.22 (1.12 to 1.33) | < 0.001 | 1.14 (1.05 to 1.24) | 0.001 |
| Haemoglobin – per 1 unit increase | 0.98 (0.89 to 1.09) | 0.722 | 0.96 (0.88 to 1.06) | 0.429 |
| Total cholesterol – per 1 unit increase | 1.00 (0.87 to 1.15) | 0.955 | 0.93 (0.82 to 1.06) | 0.269 |
| **Cardiovascular status** |  |  |  |  |
| Heart rate – per 1 bpm increase | 1.04 (1.02 to 1.05) | < 0.001 | 1.02 (1.01 to 1.03) | < 0.001 |
| **Questionnaire data** |  |  |  |  |
| SGRQ-C – per 4 point increase | 1.13 (1.10 to 1.17) | < 0.001 | 1.07 (1.03 to 1.10) | < 0.001 |
| CAT – per 1 point increase | 1.09 (1.07 to 1.11) | < 0.001 | 1.05 (1.03 to 1.07) | < 0.001 |
| **Musculoskeletal measures** |  |  |  |  |
| Six-minute walk distance – per 30 metre decrease | 1.19 (1.15 to 1.24) | < 0.001 | 1.13 (1.08 to 1.17) | < 0.001 |
| SPPB score (0-12) – per 1 point decrease | 1.14 (1.07 to 1.22) | < 0.001 | 1.08 (1.01 to 1.14) | 0.019 |
| Functional limitation (SSPB) – yes | 1.68 (1.21 to 2.33) | 0.002 | 1.22 (0.91 to 1.64) | 0.179 |
| 4MGS score (0-4) – per 1 point decrease | 1.46 (1.20 to 1.76) | < 0.001 | 1.19 (1.00 to 1.41) | 0.048 |
| Balance score (0-4) – per 1 point decrease | 1.11 (0.93 to 1.33) | 0.246 | 1.07 (0.91 to 1.25) | 0.434 |
| Chair stand score (0-4) – per 1 point decrease | 1.25 (1.11 to 1.40) | < 0.001 | 1.14 (1.02 to 1.26) | 0.016 |
| QMVC peak – per 1 kg decrease | 1.05 (1.03 to 1.07) | < 0.001 | 1.02 (1.00 to 1.03) | 0.039 |

Incidence rate ratios were estimated based on negative binomial regression. All analyses were adjusted for recruitment site.

^a^ Adjusted for age and sex

^b^ Further adjusted for body mass index, smoking status, forced expiratory volume in one second, phlegm, and exacerbation history.

^c^ P values based on negative binomial regression.

¶ Variables MRC dyspnoea score and white cell count were omitted due to collinearity.

CI = confidence intervals. FEV_1_ = forced expiratory volume in one second. GOLD = global initiative for obstructive lung disease. GFR = glomerular filtration rate. SGRQ-C = St. George respiratory questionnaire for COPD. CAT = COPD assessment test. 6MWT = six-minute walk test. SPPB = short physical performance battery. 4MGS = four-metre gait speed. QMVC = quadriceps maximum voluntary contraction.
