# Supplementary material for: Risk assessment for hospital admission in patients with COPD; a multi-centre UK prospective observational study
Source: PLoS One. 2020 Feb 10;15(2):e0228940. doi: 10.1371/journal.pone.0228940 (PMC7010290; doi:10.1371/journal.pone.0228940)
Supplement: S5 Table — (DOCX) [file pone.0228940.s007.docx]

**S5 Table. Adjusted multivariable associations with H-AECOPD length of stay.**

|  | 5 year (n = 291 individuals with H-AECOPD) | | | |
| --- | --- | --- | --- | --- |
| **Baseline Characteristics** | **Incidence risk ratio (95% CI) ^a^** | ***P* value ^c^** | **Incidence risk ratio (95% CI) ^b^** | ***P* value ^c^** |
| **Description** |  |  |  |  |
| Age – per 10 year increase | 1.78 (1.45 to 2.20) | < 0.001 | 1.83 (1.48 to 2.26) | < 0.001 |
| Sex – male | 0.84 (0.58 to 1.21) | 0.354 | 0.84 (0.56 to 1.26) | 0.399 |
| Body mass index – per 1 point increase | 0.96 (0.94 to 0.99) | 0.011 | 0.96 (0.93 to 0.99) | 0.009 |
| **Lung function** |  |  |  |  |
| FEV_1_ – per 100 ml increase | 0.96 (0.91 to 1.00) | 0.063 | 0.97 (0.93 to 1.02) | 0.269 |
| Smoking status – current | 1.39 (0.93 to 2.09) | 0.110 | 1.2 (0.78 to 1.87) | 0.409 |
| GOLD stage – per increase to next stage | 1.14 (0.86 to 1.50) | 0.374 | 1.15 (0.87 to 1.53) | 0.335 |
| Exacerbation history (1 year), ≥ 1 | 0.63 (0.41 to 0.97) | 0.035 | 0.62 (0.39 to 0.97) | 0.037 |
| Productive cough – yes | 0.75 (0.34 to 1.66) | 0.483 | 1.12 (0.77 to 1.62) | 0.559 |
| **Biochemical measures** |  |  |  |  |
| Glucose – per 1 log unit increase | 7.89 (2.67 to 23.33) | < 0.001 | 8.78 (2.81 to 27.49) | < 0.001 |
| Fibrinogen – per 1 log unit increase | 2.50 (1.11 to 5.61) | 0.027 | 3.14 (1.37 to 7.18) | 0.007 |
| CRP – per 1 log unit increase | 1.07 (0.92 to 1.24) | 0.407 | 1.14 (0.97 to 1.35) | 0.107 |
| GFR – per 1 unit increase | 0.99 (0.98 to 1.00) | 0.050 | 0.98 (0.97 to 1.00) | 0.014 |
| Neutrophils – per 1 unit increase | 1.07 (0.97 to 1.18) | 0.164 | 1.04 (0.93 to 1.16) | 0.525 |
| Haemoglobin – per 1 unit increase | 0.94 (0.83 to 1.05) | 0.273 | 0.91 (0.80 to 1.03) | 0.134 |
| Total cholesterol – per 1 unit increase | 0.93 (0.81 to 1.08) | 0.358 | 0.93 (0.79 to 1.09) | 0.349 |
| **Cardiovascular status** |  |  |  |  |
| Heart rate – per 1 bpm increase | 1.00 (0.99 to 1.02) | 0.478 | 1.00 (0.98 to 1.01) | 0.665 |
| **Questionnaire data** |  |  |  |  |
| SGRQ-C – per 4 point increase | 1.00 (0.96 to 1.03) | 0.857 | 1.02 (0.97 to 1.06) | 0.449 |
| CAT – per 1 point increase | 0.99 (0.97 to 1.01) | 0.504 | 1.00 (0.98 to 1.03) | 0.892 |
| **Musculoskeletal measures** |  |  |  |  |
| Six-minute walk distance – per 30 metre decrease | 1.11 (1.05 to 1.16) | < 0.001 | 1.14 (1.08 to 1.20) | < 0.001 |
| SPPB score (0-12) – per 1 point decrease | 1.15 (1.06 to 1.24) | < 0.001 | 1.18 (1.10 to 1.27) | < 0.001 |
| Functional limitation (SSPB) – yes | 1.84 (1.27 to 2.68) | 0.001 | 2.01 (1.37 to 2.94) | < 0.001 |
| 4MGS score (0-4) – per 1 point decrease | 1.29 (1.01 to 1.65) | 0.045 | 1.31 (1.03 to 1.67) | 0.029 |
| Balance score (0-4) – per 1 point decrease | 1.44 (1.12 to 1.84) | 0.004 | 1.45 (1.13 to 1.86) | 0.003 |
| Chair stand score (0-4) – per 1 point decrease | 1.24 (1.09 to 1.40) | 0.001 | 1.32 (1.16 to 1.49) | < 0.001 |
| QMVC peak – per 1 kg decrease | 1.03 (1.01 to 1.05) | 0.002 | 1.02 (1.00 to 1.04) | 0.056 |

Incidence rate ratios were estimated based on negative binomial regression. All analyses were adjusted for recruitment site.

^a^ Adjusted for age and sex

^b^ Further adjusted for body mass index, smoking status, forced expiratory volume in one second, phlegm, and exacerbation history.

^c^ P values based on negative binomial regression.

¶ Variables MRC dyspnoea score and white cell count were omitted due to collinearity.

CI = confidence intervals. FEV_1_ = forced expiratory volume in one second. GOLD = global initiative for obstructive lung disease. GFR = glomerular filtration rate. SGRQ-C = St. George respiratory questionnaire for COPD. CAT = COPD assessment test. 6MWT = six-minute walk test. SPPB = short physical performance battery. 4MGS = four-metre gait speed. QMVC = quadriceps maximum voluntary contraction.
