# Supplementary material for: Risk assessment for hospital admission in patients with COPD; a multi-centre UK prospective observational study
Source: PLoS One. 2020 Feb 10;15(2):e0228940. doi: 10.1371/journal.pone.0228940 (PMC7010290; doi:10.1371/journal.pone.0228940)
Supplement: S6 Table — (DOCX) [file pone.0228940.s008.docx]

**S6 Table. Adjusted multivariate associations with H-AECOPD rate, by exacerbation history.**

|  | 5 year (n = 291 individuals with H-AECOPD) | | | |
| --- | --- | --- | --- | --- |
|  | Exacerbation history (n = 473) | | No exacerbation history (n = 236) | |
| **Baseline Characteristics** | **Incidence risk ratio (95% CI) ^a^** | ***P* value ^b^** | **Incidence risk ratio (95% CI) ^a^** | ***P* value ^b^** |
| **Description** |  |  |  |  |
| Age – per 10 year increase | 0.90 (0.74 to 1.09) | 0.283 | 0.69 (0.47 to 1.02) | 0.063 |
| Sex – male | 2.05 (1.46 to 2.89) | < 0.001 | 5.39 (2.55 to 11.41) | < 0.001 |
| Body mass index – per 1 point increase | 0.98 (0.96 to 1.01) | 0.128 | 1.08 (1.02 to 1.15) | 0.010 |
| **Lung function** |  |  |  |  |
| FEV_1_ – per 100 ml increase | 0.85 (0.82 to 0.88) | < 0.001 | 0.80 (0.75 to 0.86) | < 0.001 |
| Smoking status – current | 1.07 (0.75 to 1.52) | 0.723 | 1.10 (0.58 to 2.11) | 0.762 |
| GOLD stage – per increase to next stage | 2.17 (1.73 to 2.74) | < 0.001 | 3.74 (2.36 to 5.93) | < 0.001 |
| Exacerbation history (1 year), ≥ 1 |  |  |  |  |
| Productive cough – yes | 1.14 (0.84 to 1.56) | 0.408 | 1.23 (0.63 to 2.42) | 0.539 |
| **Biochemical measures** |  |  |  |  |
| Glucose – per 1 log unit increase | 1.71 (0.57 to 5.10) | 0.337 | 2.50 (0.36 to 17.33) | 0.353 |
| Fibrinogen – per 1 log unit increase | 1.98 (0.99 to 3.97) | 0.055 | 1.96 (0.44 to 8.60) | 0.375 |
| CRP – per 1 log unit increase | 1.18 (1.03 to 1.35) | 0.018 | 0.90 (0.66 to 1.22) | 0.500 |
| GFR – per 1 unit increase | 1.00 (0.99 to 1.01) | 0.458 | 0.99 (0.97 to 1.01) | 0.264 |
| Neutrophils – per 1 unit increase | 1.15 (1.06 to 1.26) | 0.002 | 1.07 (0.88 to 1.30) | 0.518 |
| Haemoglobin – per 1 unit increase | 0.95 (0.86 to 1.05) | 0.302 | 1.12 (0.88 to 1.41) | 0.358 |
| Total cholesterol – per 1 unit increase | 0.95 (0.83 to 1.10) | 0.497 | 1.01 (0.72 to 1.40) | 0.969 |
| **Cardiovascular status** |  |  |  |  |
| Heart rate – per 1 bpm increase | 1.02 (1.01 to 1.03) | 0.003 | 1.02 (0.99 to 1.05) | 0.122 |
| **Questionnaire data** |  |  |  |  |
| SGRQ-C – per 4 point increase | 1.07 (1.03 to 1.11) | 0.001 | 1.07 (1.00 to 1.15) | 0.037 |
| CAT – per 1 point increase | 1.04 (1.02 to 1.06) | < 0.001 | 1.07 (1.02 to 1.11) | 0.004 |
| **Musculoskeletal measures** |  |  |  |  |
| Six-minute walk distance – per 30 metre decrease | 1.11 (1.06 to 1.16) | < 0.001 | 1.16 (1.06 to 1.26) | 0.001 |
| SPPB score (0-12) – per 1 point decrease | 1.11 (1.04 to 1.19) | 0.003 | 0.94 (0.80 to 1.10) | 0.435 |
| Functional limitation (SSPB) – yes | 1.40 (1.01 to 1.95) | 0.046 | 0.81 (0.43 to 1.54) | 0.524 |
| 4MGS score (0-4) – per 1 point decrease | 1.28 (1.07 to 1.54) | 0.008 | 0.82 (0.48 to 1.42) | 0.476 |
| Balance score (0-4) – per 1 point decrease | 1.13 (0.95 to 1.35) | 0.175 | 0.71 (0.45 to 1.12) | 0.140 |
| Chair stand score (0-4) – per 1 point decrease | 1.18 (1.05 to 1.33) | 0.006 | 1.02 (0.82 to 1.28) | 0.827 |
| QMVC peak – per 1 kg decrease | 1.02 (1.00 to 1.03) | 0.085 | 1.00 (0.97 to 1.04) | 0.895 |

Incidence rate ratios were estimated based on negative binomial regression. All analyses were stratified by recruitment centre and exacerbation history.

^a^ Adjusted for age, sex, body mass index, smoking status, forced expiratory volume in one second, and phlegm.

^c^ P values based on negative binomial regression.

¶ Variables MRC dyspnoea score and white cell count were omitted due to collinearity.

CI = confidence intervals. FEV_1_ = forced expiratory volume in one second. GOLD = global initiative for obstructive lung disease. GFR = glomerular filtration rate. SGRQ-C = St. George respiratory questionnaire for COPD. CAT = COPD assessment test. 6MWT = six-minute walk test. SPPB = short physical performance battery. 4MGS = four-metre gait speed. QMVC = quadriceps maximum voluntary contraction.
