# Supplementary material for: Risk assessment for hospital admission in patients with COPD; a multi-centre UK prospective observational study
Source: PLoS One. 2020 Feb 10;15(2):e0228940. doi: 10.1371/journal.pone.0228940 (PMC7010290; doi:10.1371/journal.pone.0228940)
Supplement: S7 Table — (DOCX) [file pone.0228940.s009.docx]

**S7 Table. Adjusted multivariate associations with H-AECOPD rate, by sex.**

|  | 5 year (n = 291 individuals with H-AECOPD) | | | |
| --- | --- | --- | --- | --- |
|  | Male (n = 434) | | Female (n = 280) | |
| **Baseline Characteristics** | **Incidence risk ratio (95% CI) ^a^** | ***P* value ^b^** | **Incidence risk ratio (95% CI) ^a^** | ***P* value ^b^** |
| **Description** |  |  |  |  |
| Age – per 10 year increase | 0.89 (0.72 to 1.11) | 0.313 | 0.77 (0.58 to 1.02) | 0.073 |
| Sex – male |  |  |  |  |
| Body mass index – per 1 point increase | 1.02 (0.99 to 1.06) | 0.237 | 0.98 (0.95 to 1.01) | 0.242 |
| **Lung function** |  |  |  |  |
| FEV_1_ – per 100 ml increase | 0.84 (0.81 to 0.87) | < 0.001 | 0.81 (0.76 to 0.87) | < 0.001 |
| Smoking status – current | 0.87 (0.57 to 1.32) | 0.502 | 1.51 (0.96 to 2.36) | 0.073 |
| GOLD stage – per increase to next stage | 2.87 (2.21 to 3.73) | < 0.001 | 2.01 (1.41 to 2.87) | < 0.001 |
| Exacerbation history (1 year), ≥ 1 | 1.50 (1.03 to 2.18) | 0.033 | 3.45 (1.78 to 6.67) | < 0.001 |
| Productive cough – yes | 1.05 (0.72 to 1.51) | 0.810 | 1.19 (0.78 to 1.82) | 0.409 |
| **Biochemical measures** |  |  |  |  |
| Glucose – per 1 log unit increase | 0.66 (0.19 to 2.31) | 0.516 | 6.67 (1.68 to 26.56) | 0.007 |
| Fibrinogen – per 1 log unit increase | 1.42 (0.61 to 3.3) | 0.412 | 2.96 (1.08 to 8.14) | 0.035 |
| CRP – per 1 log unit increase | 1.08 (0.92 to 1.26) | 0.373 | 1.18 (0.97 to 1.45) | 0.104 |
| GFR – per 1 unit increase | 1.00 (0.99 to 1.01) | 0.690 | 1.00 (0.99 to 1.02) | 0.947 |
| Neutrophils – per 1 unit increase | 1.01 (0.90 to 1.13) | 0.899 | 1.30 (1.16 to 1.46) | < 0.001 |
| Haemoglobin – per 1 unit increase | 0.96 (0.86 to 1.08) | 0.525 | 0.93 (0.78 to 1.10) | 0.383 |
| Total cholesterol – per 1 unit increase | 0.93 (0.79 to 1.10) | 0.403 | 0.93 (0.76 to 1.13) | 0.469 |
| **Cardiovascular status** |  |  |  |  |
| Heart rate – per 1 bpm increase | 1.01 (1.00 to 1.03) | 0.082 | 1.03 (1.01 to 1.05) | 0.001 |
| **Questionnaire data** |  |  |  |  |
| SGRQ-C – per 4 point increase | 1.06 (1.02 to 1.11) | 0.004 | 2.40 (1.54 to 3.74) | < 0.001 |
| CAT – per 1 point increase | 1.05 (1.02 to 1.08) | < 0.001 | 1.06 (1.01 to 1.11) | 0.023 |
| **Musculoskeletal measures** |  |  |  |  |
| Six-minute walk distance – per 30 metre decrease | 1.12 (1.07 to 1.18) | < 0.001 | 1.04 (1.01 to 1.08) | 0.005 |
| SPPB score (0-12) – per 1 point decrease | 1.03 (0.94 to 1.12) | 0.546 | 1.15 (1.05 to1.26) | 0.002 |
| Functional limitation (SSPB) – yes | 1.03 (0.71 to 1.50) | 0.876 | 1.70 (1.05 to 2.73) | 0.029 |
| 4MGS score (0-4) – per 1 point decrease | 1.09 (0.84 to 1.40) | 0.527 | 1.40 (1.10 to 1.78) | 0.007 |
| Balance score (0-4) – per 1 point decrease | 0.95 (0.71 to 1.27) | 0.732 | 1.11 (0.91 to 1.35) | 0.303 |
| Chair stand score (0-4) – per 1 point decrease | 1.06 (0.93 to 1.21) | 0.378 | 1.35 (1.13 to 1.61) | 0.001 |
| QMVC peak – per 1 kg decrease | 1.02 (1.00 to 1.04) | 0.048 | 1.03 (1.00 to 1.06) | 0.082 |

Incidence rate ratios were estimated based on negative binomial regression. Analyses were stratified by recruitment centre and sex.

^a^ Adjusted for age, body mass index, smoking status, forced expiratory volume in one second, phlegm, and exacerbation history.

^b^ P values based on negative binomial regression.

¶ Variables MRC dyspnoea score and white cell count were omitted due to collinearity.

CI = confidence intervals. FEV_1_ = forced expiratory volume in one second. GOLD = global initiative for obstructive lung disease. GFR = glomerular filtration rate. SGRQ-C = St. George respiratory questionnaire for COPD. CAT = COPD assessment test. 6MWT = six-minute walk test. SPPB = short physical performance battery. 4MGS = four-metre gait speed. QMVC = quadriceps maximum voluntary contraction.
