# Supplementary figures and images for: Risk assessment for hospital admission in patients with COPD; a multi-centre UK prospective observational study
Source: PLoS One. 2020 Feb 10;15(2):e0228940. doi: 10.1371/journal.pone.0228940 (PMC7010290; doi:10.1371/journal.pone.0228940)

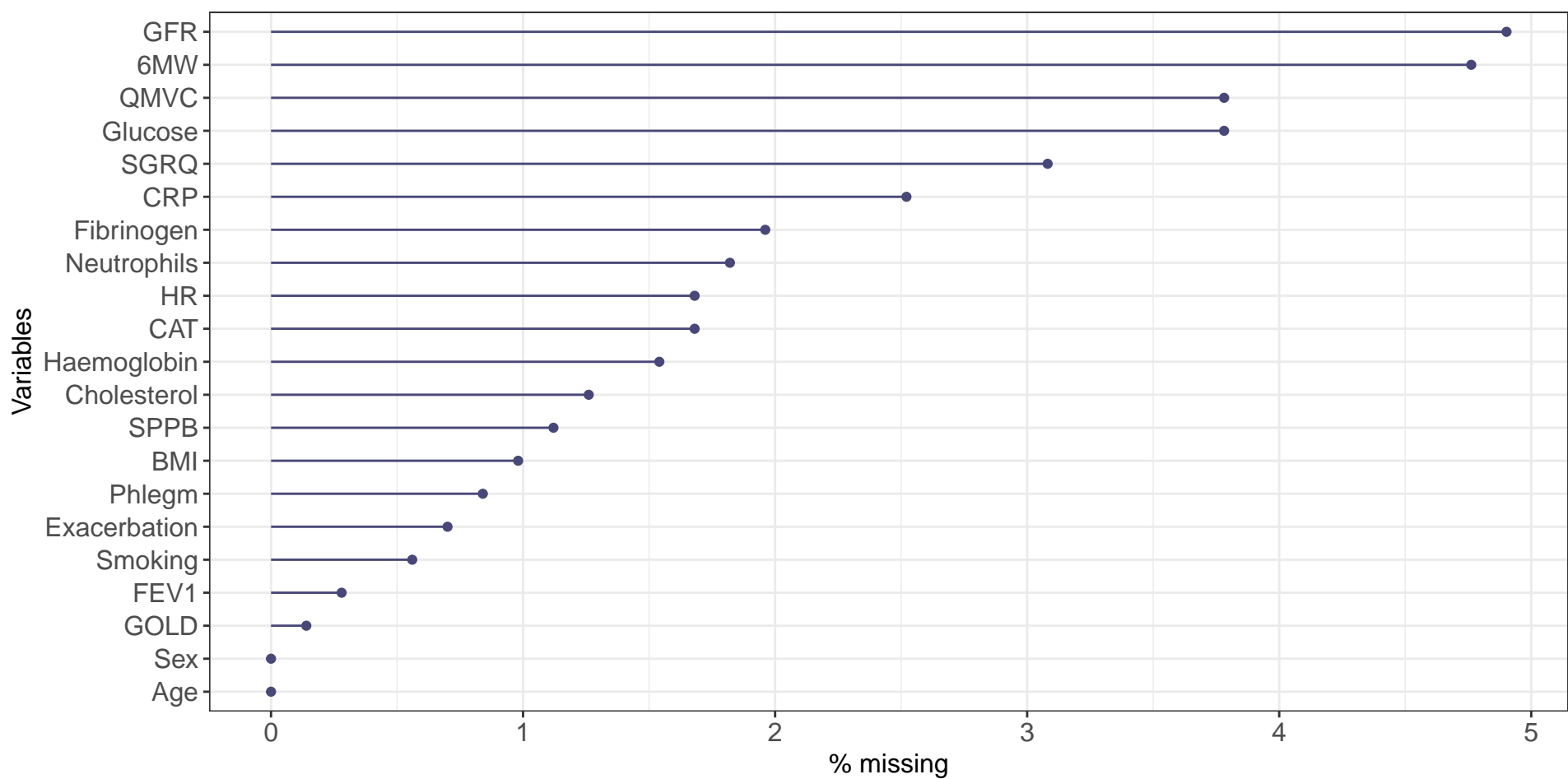

Supplement: S1 Fig — GFR = glomerular filtration rate. 6MW = six-minute walk. QMVC = quadriceps maximum voluntary contraction. CRP = C-reactive protein. HR = heart rate. CAT = COPD assessment test. SPPB = short physical performance battery. BMI = body mass index. FEV1 = forced expiratory volume in one second. GOLD = global initiative for obstructive lung disease. (PDF) [file pone.0228940.s010.pdf]

Intersection Size

20

10

0

SGRQ\_NA

Glucose\_NA

QMVC\_NA

6MW\_NA

GFR\_NA

24

24

19

18

16

4

4

2

2

1

1

1

1

1

1

30

20

10

0

Set Size

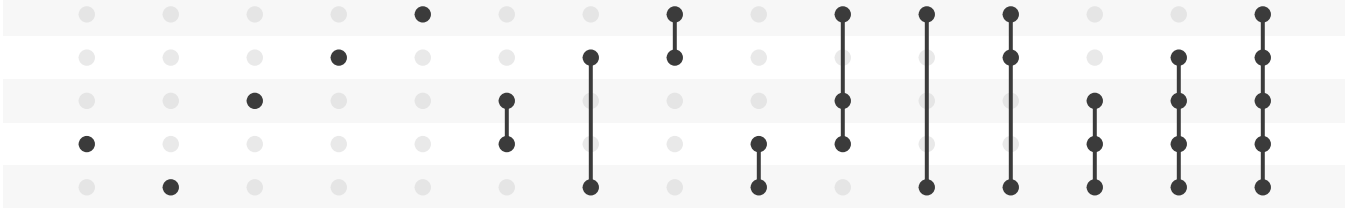

Supplement: S2 Fig — SGRQ = St. George respiratory questionnaire for COPD. QMVC = quadriceps maximum voluntary contraction. 6MW = six-minute walk. GFR = glomerular filtration rate. (PDF) [file pone.0228940.s011.pdf]

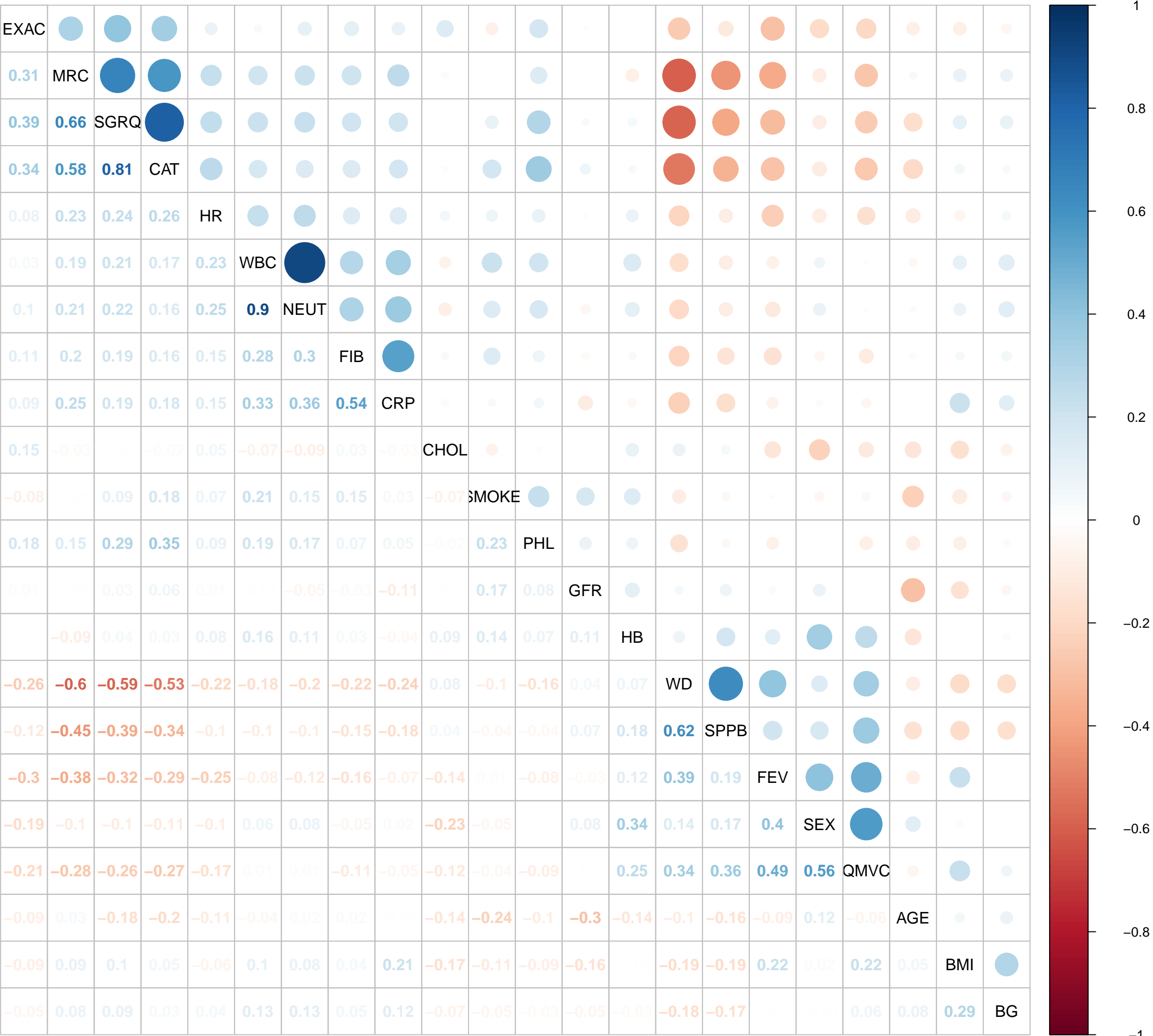

Supplement: S3 Fig — EXAC = exacerbation history. MRC = Medical Research Council dyspnoea score. SGRQ = St. George respiratory questionnaire for COPD. CAT = COPD assessment test. HR = heart rate. WBC = white cell count. NEUT = neutrophils. FIB = fibrinogen. CRP = C-reactive protein. CHOL = total cholesterol. SMOKE = smoking status. PHL = phlegm. GRF = glomerular filtration rate. HB = haemoglobin. WD = six-minute walk. SPPB = short physical performance battery. FEV = forced expiratory volume in one second. QMVC = quadriceps maximum voluntary contraction. BMI = body mass index. BG = glucose. Correlation coefficients with a values <0.30 were considered weak, 0.30–0.50 as moderate, and >0.50 as strong. (PDF) [file pone.0228940.s012.pdf]

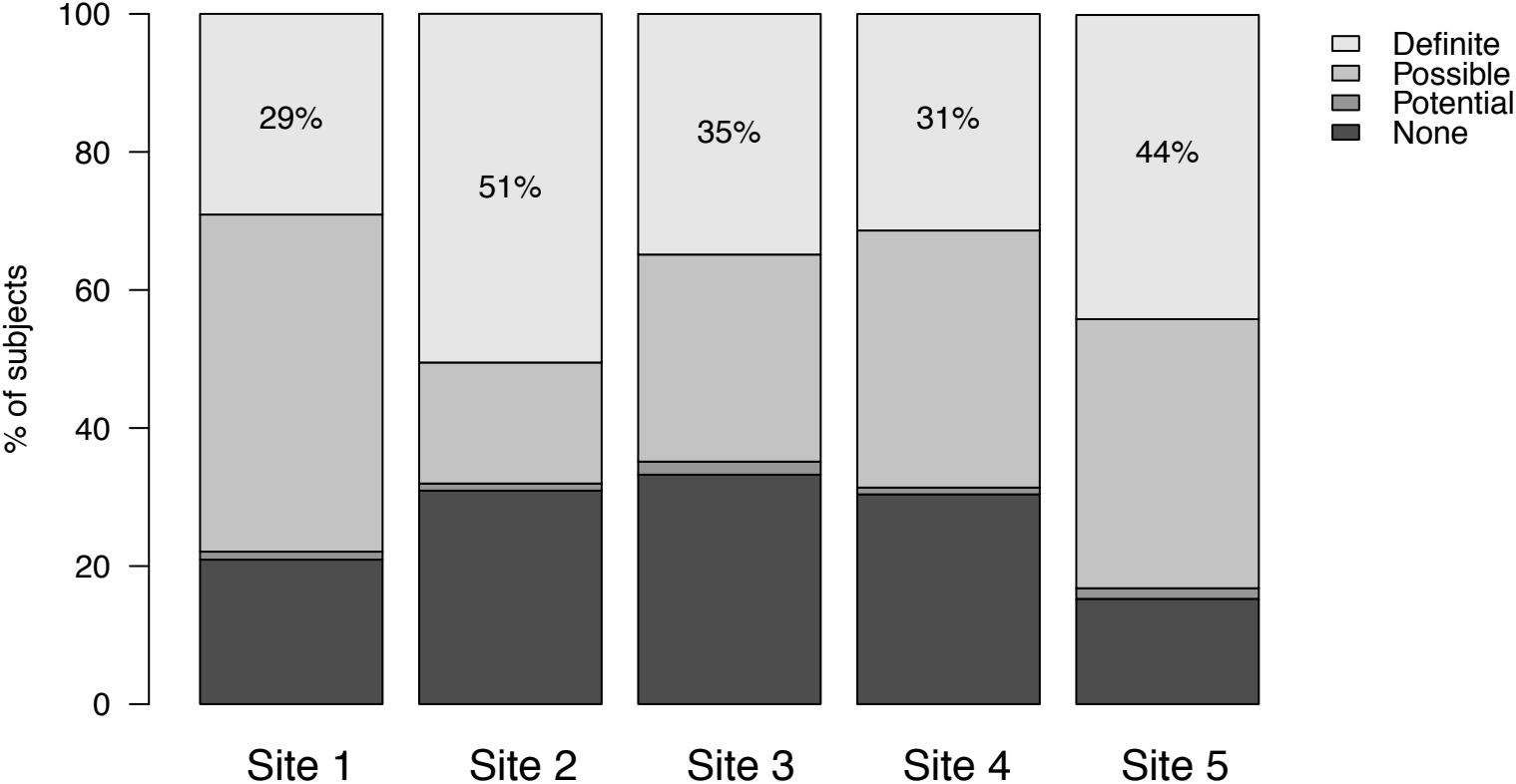

Supplement: S5 Fig — Hospital admission data obtained from the National Health Service (NHS) Digital, NHS Wales, and NHS Scotland. (PDF) [file pone.0228940.s014.pdf]

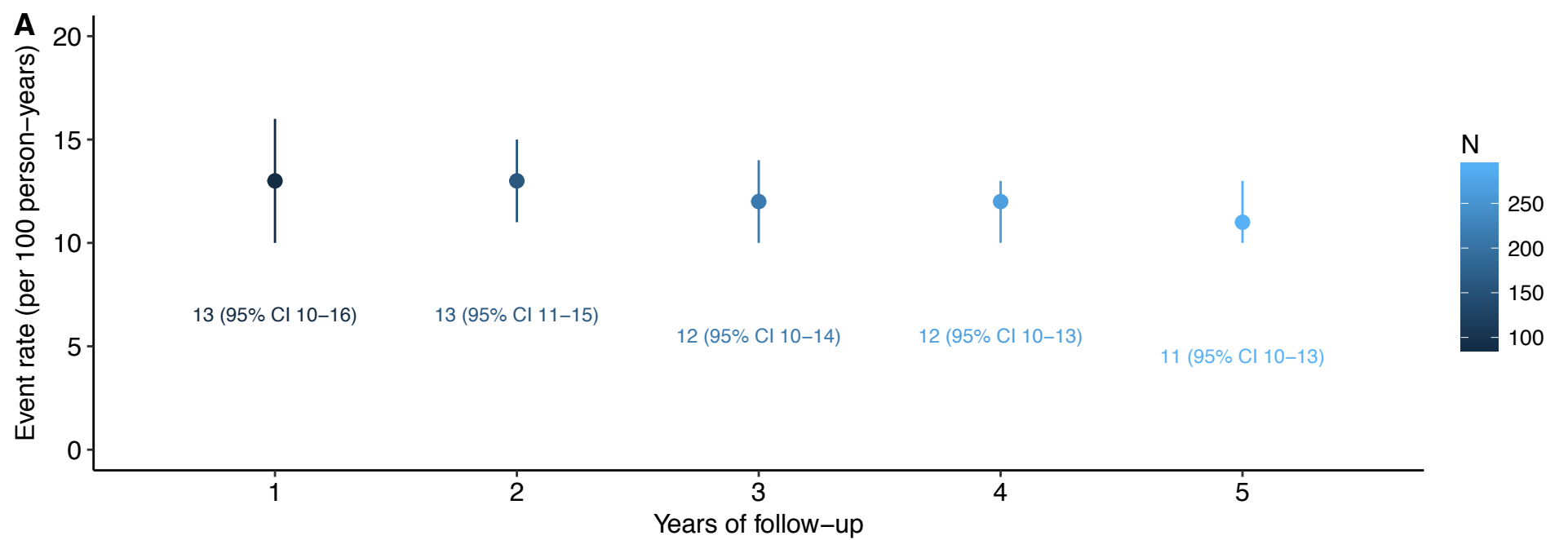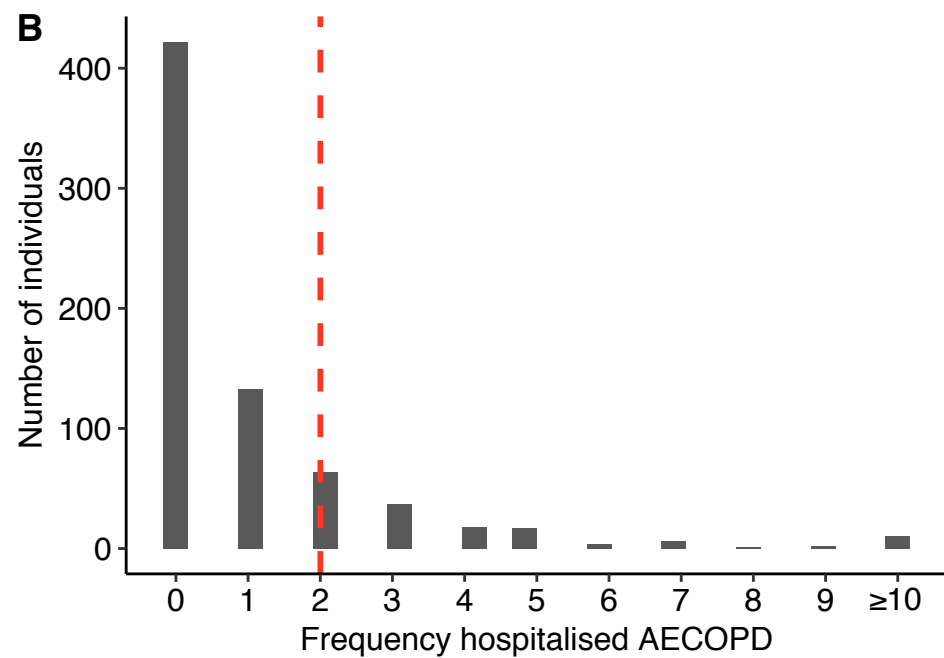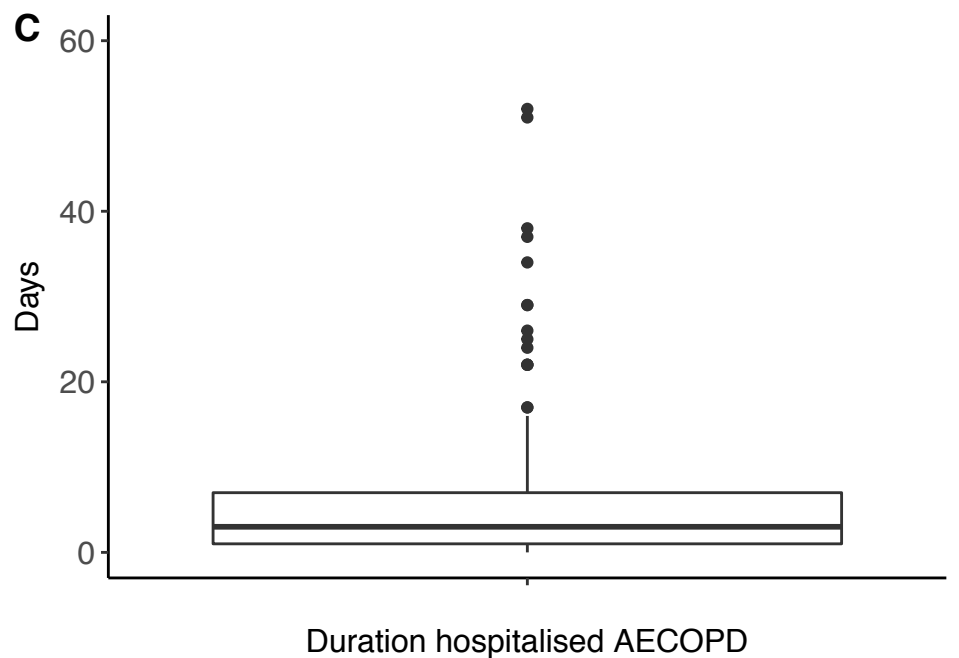

Supplement: S6 Fig — (A) Mean event rates with 95% confidence intervals per 100 per-years during study period, (B) H-AECOPD frequency, and (C) H-AECOPD duration. Depth of blue indicates the cumulative number of individuals with first H-AECOPD during the study period. Red dashed line indicates the median number of hospital admissions for H-AECOPD amongst those experienced an H-AECOPD. (PDF) [file pone.0228940.s015.pdf]

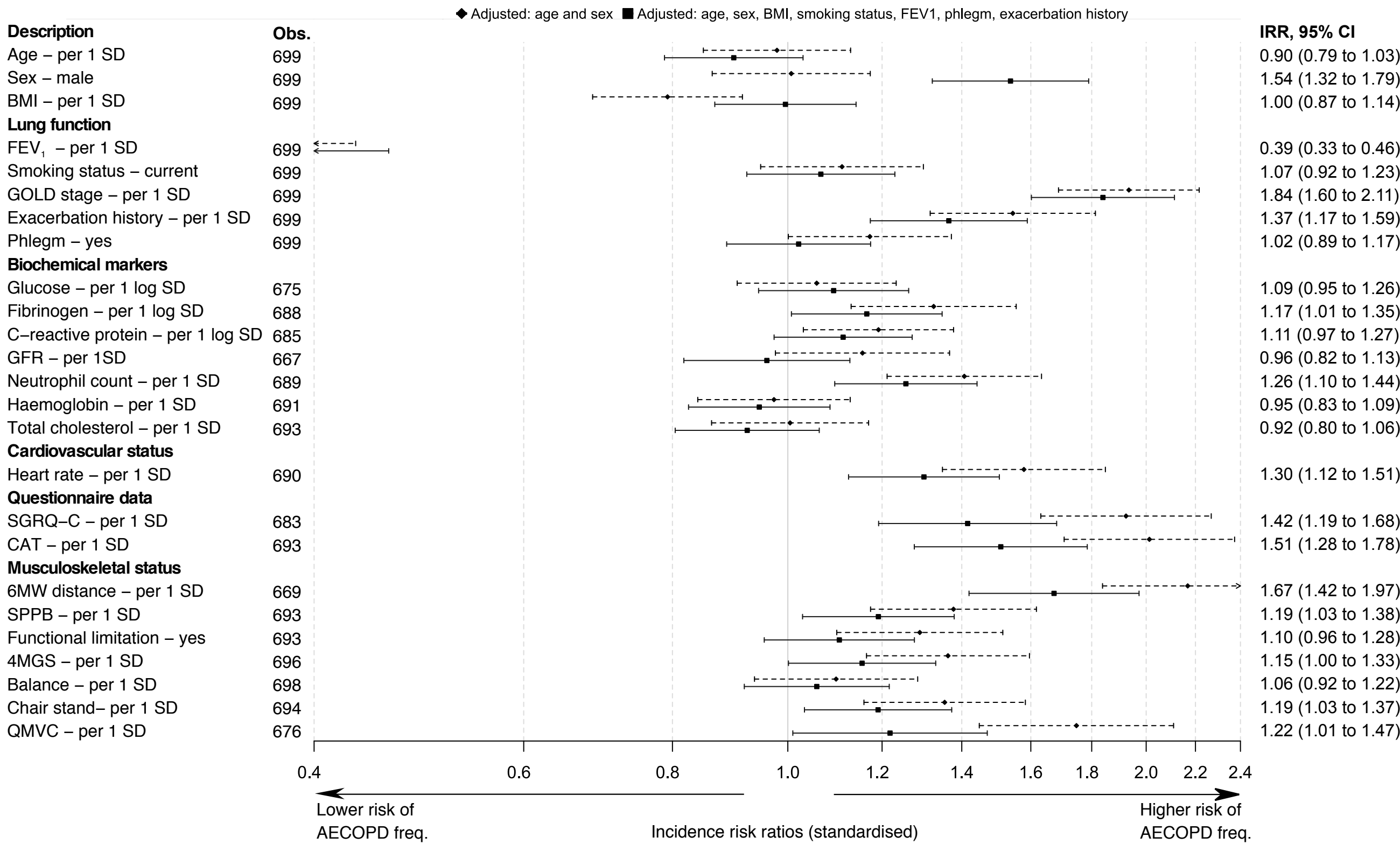

Supplement: S7 Fig — (PDF) [file pone.0228940.s016.pdf]

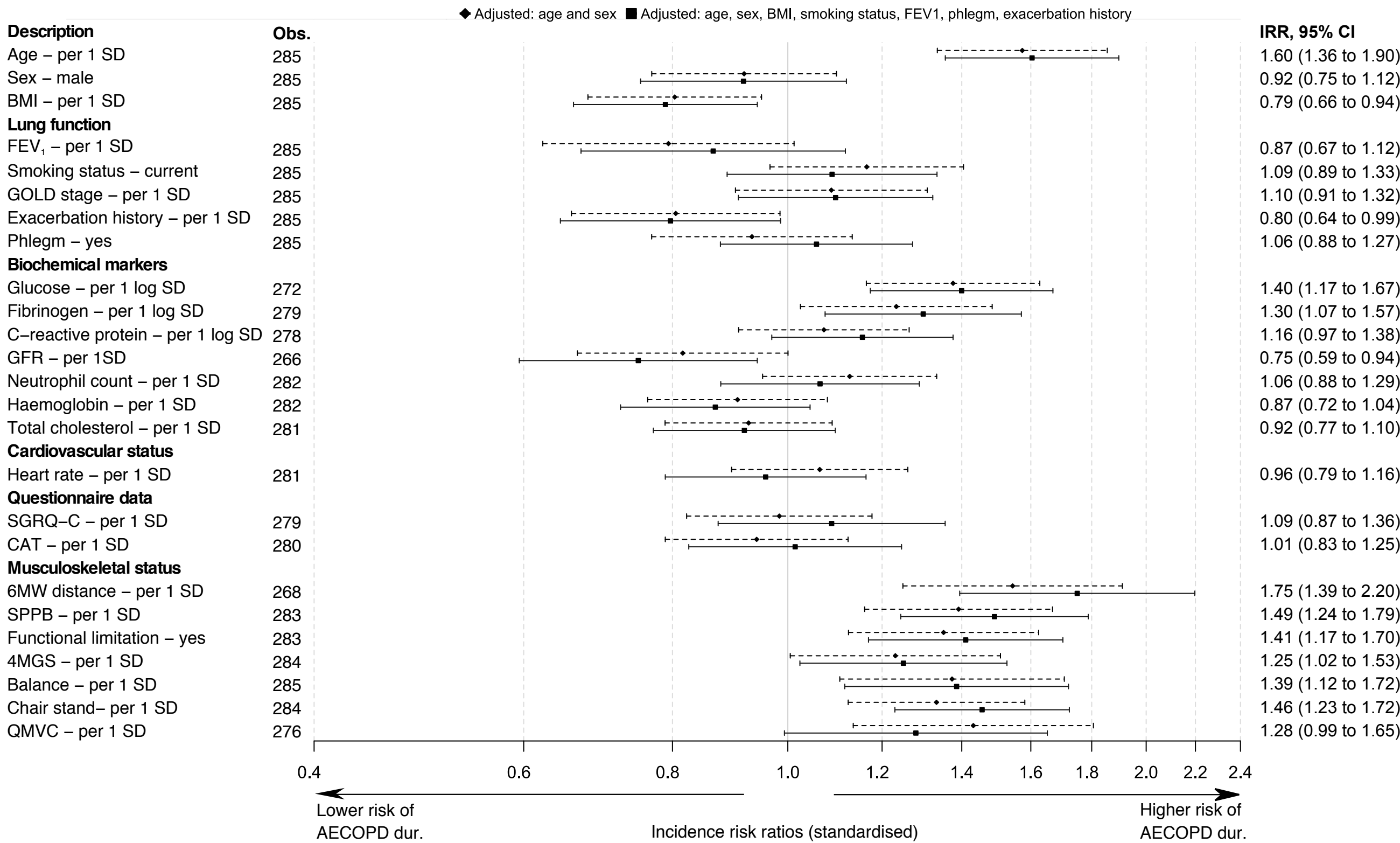

Supplement: S8 Fig — (PDF) [file pone.0228940.s017.pdf]
