# Supplementary material for: Risk assessment for hospital admission in patients with COPD; a multi-centre UK prospective observational study
Source: PLoS One. 2020 Feb 10;15(2):e0228940. doi: 10.1371/journal.pone.0228940 (PMC7010290; doi:10.1371/journal.pone.0228940)
Supplement: S4 Fig — Total number of H-AECOPD (n = 291). FEV1 = forced expiratory volume in one second. FVC = forced vital capacity. NHS = National Health Services. H-AECOPD = hospitalised acute exacerbation of COPD. FUP = follow-up period. (DOCX) [file pone.0228940.s013.docx]

Individuals screened (n = 746)

## Screening

Did not meet inclusion criteria (n = 17)

Individuals entered into study (n = 729)

## Study entry

Individuals completed visit (n = 729)

## Baseline visit

Not flagged by NHS (n = 15)

Individuals with H-AECOPD status (n = 714)

## Flagging

Individuals included in analysis (n = 714)

## Analysis

H-AECOPD (n = 291)

No

H-AECOPD (n = 423)

## FUP 5-yrs
